# A Frog Skin‐Derived Peptide Targeting SCD1 Exerts Radioprotective Effects Against Skin Injury by Inhibiting STING‐Mediated Inflammation

**DOI:** 10.1002/advs.202306253

**Published:** 2024-04-06

**Authors:** Fenghao Geng, Li Zhong, Tingyi Yang, Jianhui Chen, Ping Yang, Fengdi Jiang, Tao Yan, Bin Song, Zuxiang Yu, Daojiang Yu, Jie Zhang, Jianping Cao, Shuyu Zhang

**Affiliations:** ^1^ Laboratory of Radiation Medicine West China School of Basic Medical Sciences & Forensic Medicine Sichuan University Chengdu 610041 China; ^2^ Laboratory of Radiation Medicine West China Second University Hospital Sichuan University Chengdu 610041 China; ^3^ Radiation Medicine Department of Institute of Preventive Medicine Fourth Military Medical University Xi'an 710032 China; ^4^ School of Radiation Medicine and Protection State Key Laboratory of Radiation Medicine Soochow University Suzhou 215123 China; ^5^ The Second Affiliated Hospital of Chengdu Medical College China National Nuclear Corporation 416 Hospital Chengdu 610051 China; ^6^ NHC Key Laboratory of Nuclear Technology Medical Transformation (Mianyang Central Hospital) Mianyang 621099 China

**Keywords:** frog, ionizing radiation, peptide, radioprotection, stearyl‐CoA desaturase 1 (SCD1)

## Abstract

The extensive application of nuclear technology has increased the potential of uncontrolled radiation exposure to the public. Since skin is the largest organ, radiation‐induced skin injury remains a serious medical concern. Organisms evolutionally develop distinct strategies to protect against environment insults and the related research may bring novel insights into therapeutics development. Here, 26 increased peptides are identified in skin tissues of frogs (*Pelophylax nigromaculatus*) exposed to electron beams, among which four promoted the wound healing of irradiated skin in rats. Specifically, radiation‐induced frog skin peptide‐2 (RIFSP‐2), from histone proteolysis exerted membrane permeability property, maintained cellular homeostasis, and reduced pyroptosis of irradiated cells with decreased TBK1 phosphorylation. Subsequently, stearyl‐CoA desaturase 1 (SCD1) is identified, a critical enzyme in biogenesis of monounsaturated fatty acids (MUFAs) as a direct target of RIFSP‐2 based on streptavidin‐biotin system. The lipidomic analysis further assured the restrain of MUFAs biogenesis by RIFSP‐2 following radiation. Moreover, the decreased MUFA limited radiation‐induced and STING‐mediated inflammation response. In addition, genetic depletion or pharmacological inhibition of STING counteracted the decreased pyroptosis by RIFSP‐2 and retarded tissue repair process. Altogether, RIFSP‐2 restrains radiation‐induced activation of SCD1‐MUFA‐STING axis. Thus, the stress‐induced amphibian peptides can be a bountiful source of novel radiation mitigators.

## Graphical abstract

Radiation‐induced skin injury remains a serious medical concern. The radiation induced frog skin peptide‐2 (RIFSP‐2), identified in this study exerts membrane permeability property and accelerates tissue repair process of irradiated skin tissues. Mechanically, RIFSP‐2 directly interacts with SCD1 and restrains biogenesis of MUFAs, which further limits the STING‐mediated phosphorylation of TBK1 and subsequently the inflammatory cell injury of irradiated cells.

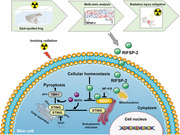

## 1 Introduction

Ionizing radiation has been extensively used in the military, medical, scientific, and industrial practice. Terrorism attacks with radiological devices, nuclear power facility disasters, potential nuclear wars, or occupational exposure are common causes of radiation injury.^[^ ^1^, ^2^, ^3^, ^4^ ^]^ Lying in the first line against external insults, skin is the largest organ and relatively sensitive to radiation. And for this reason, the prevention and management of radiation‐induced skin injury occupies in the nuclear and radiological emergency preparedness and response. Besides, in medical treatment, radiotherapy is one of the major approaches for treating cancers. Nevertheless, ≈ 95% of patients are estimated to suffer from radiation‐induced skin injury.^[^ ^5^, ^6^ ^]^ This disease remains a serious concern because no effective countermeasures have been developed yet. Free radicals generated by ionizing radiation directly destroy DNA and other macromolecular components in skin cells. In conjunction, the local inflammatory reaction primarily mediates pathogenesis of radiation‐induced skin injury, however the details remain to be elucidated.^[^ ^7^ ^]^ Distinct from mechanical or chemical injury, radiation‐induced skin injury is likely to be recurrent and aggravates throughout treatment.^[^ ^8^ ^]^ Although there has been an enormous global effort to develop treatments against radiation injury over the past 70 years, with much attention on fatal hematopoietic or gastrointestinal radiation injury, radiation‐induced skin injury remains to be clinically irradicable.^[^ ^9^ ^]^ Development of novel and effective agents against radiation‐induced skin injury remain a persistent unmet medical need that increases life expectancy and quality of life.

In response to adverse conditions such as ionizing radiation, both prokaryotic and eukaryotic organisms have evolutionally developed specific strategies to protect against potential injury. *Deinococcus radiodurans*, a consummate prokaryote with extreme radiation resistance, has developed unique systems to maintain high concentrations of Mn (II) to prevent protein oxidation and ensure sufficient repair enzymes to facilitate DNA damage repair.^[^ ^10^ ^]^ A DNA‐associated protein named damage suppressor protein (*DSUP*) from tardigrades (*Ramazzottius varieornatus*), a small aquatic animal that exhibits extraordinary tolerance to various physical extremes, was reported to be responsible for its radiation tolerance, as it facilitates DNA damage repair and improves the viability of irradiated human cells.^[^ ^11^ ^]^ To occupy both the terrestrial and the aquatic environments, with abundant environmental insults, the skin of amphibians not only provides a barrier against external stresses but also functions as a respiratory organ and is involved in osmoregualtoin and thermoregulation.^[^ ^12^ ^]^ Since 2015, a variety of frog‐derived peptides (FDPs), secreted by the granular glands of various frogs in the dermis, have been discovered and reported to exert versatile functions, including antioxidant, antimicrobial, anti‐inflammatory, and wound healing‐facilitating functions, making FDPs potential candidates for the development of novel pharmacological agents targeting radiation‐induced tissue injury.^[^ ^13^ ^]^ For instance, the fractionation of *Odorrana andersonii* frog skin secretion was recently reported to decrease miR‐663a levels and subsequently activate the TGF‐β/Smad signaling pathway, which accelerated skin wound re‐epithelialization and granular tissue formation.^[^ ^14^ ^]^ In addition, an immunomodulatory peptide derived from the Chinese concave‐eared frog *Odorrana tormota* directly stimulated the production of regulatory factors in macrophages and further enhanced the crosstalk between macrophages and keratinocytes/fibroblasts, which significantly promoted the healing of full‐thickness wounds.^[^ ^15^ ^]^ Although, amphibians are widely used to investigate radiation pollution in nuclear accident plants, there is rarely intensive study on alterations in frog skin tissues exposed to ionizing radiation.^[^ ^16^ ^]^ Previously, to overcome the obstacle of membrane impermeability, we previously fused the *Rana pleurade* skin‐derived antioxidant peptide with a classical HIV‐TAT protein transduction domain, and the reconstructed peptide alleviated radiation‐induced skin injury.^[^ ^17^ ^]^ Thus, investigations on the survival mechanisms of organisms during radiation can help elucidate how to overcome adverse clinical outcomes.

The stimulator of interferon genes (STING) has been reported to be a gatekeeper for cellular stress caused by ionizing radiation,^[^ ^18^ ^]^ which is activated by emerging self or extrinsic DNA recognized by cGAS, or by RNA sensed by MAVS, RIG‐I, and MDA‐5 in the cytoplasm. Previously, we noted the dysregulation of transcripts implicated in the innate immune response in irradiated human skin keratinocytes through RNA‐Seq of global genes.^[^ ^19^ ^]^ Since then, we further identified changes in genes enriched in immune or inflammatory responses in irradiated cells and observed the formation of cytosolic dsDNA, which is recognized by cGAS, and the aggregates of STING in irradiated human skin fibroblasts.^[^ ^20^ ^]^ Besides, radiation‐induced mice skin ulcers were also reported to be related with the hyperactivation of cGAS‐STING pathway.^[^ ^21^ ^]^ Most recently, we summarized the role of STING signaling in radiation‐induced tissue fibrosis.^[^ ^22^ ^]^ Therefore, STING engages in both the radiation‐induced acute skin damage and the chronic fibrosis of irradiated skin tissues. And agents targeting disturb of STING activation may shed light on prevention of the radiation‐induced skin injury. Here, through multi‐omic analysis and functional studies, we discovered considerable alterations in proteolysis‐originated polypeptides in irradiated frog skin tissues and identified one polypeptide with suitable membrane permeability named RIFSP‐2. Our results suggest that RIFSP‐2 targets SCD1‐STING axis and restrains the biogenesis of monounsaturated fatty acids (MUFAs), which maintained cell homeostasis and protected against radiation‐induced cell death.

## 2 Results

### 2.1 Frog Skin is Resistant to Ionizing Radiation

**Figure 1:**
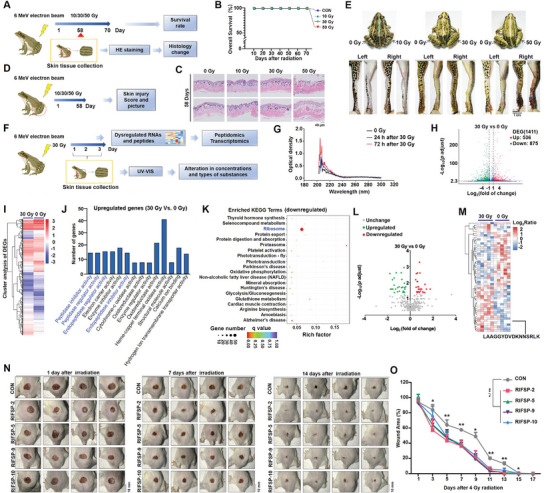
Identification of radiation‐induced frog skin peptides (RIFSPs) as mitigators of radiation‐induced skin injury. A) Schematic showing the workflow for establishing radiation‐induced skin injury on the backs and D) limbs of frogs. B) Survival curve of frogs exposed to different doses of irradiation on the back. C) Different morphological changes were detected by hematoxylin and eosin (H&E) staining of irradiated frog skin tissues on the back. E) Distinct pathological manifestations in the limb skin tissues exposed to different doses of radiation. F) Schematic showing the workflow for investigating the crude extract of skin tissues using ultraviolet‐visible absorption spectrometry (UV–VIS) and subsequently conducting RNA sequencing (RNA‐Seq) and peptidomic analysis of irradiated frog skin tissues. G) Alterations in the optical density of irradiated frog skin tissue crude extracts. Volcano plot of the differentially expressed genes H) and peptides L) in irradiated frog skin tissues. Cluster analysis I), Gene Ontology (GO) analysis, J) and Kyoto Encyclopedia of Genes and Genomes K) analysis of the differentially expressed genes induced by irradiation. M) Cluster analysis of differentially expressed peptides. Pictures of typical combined skin injury after irradiation N) and wound healing O) in the combined skin injury model mice treated with different RIFSPs. *P < 0.05 and **P < 0.01, compared with the control group.

Frog skins have been reported to be resistant to a variety of stresses, including UV radiation.^[^ ^23^, ^24^ ^]^ To verify the resistance of frog skin tissues to ionizing radiation, we first established a back skin irradiation model with a 6 MeV electron beam (**Figure** **1**A). Briefly, frog skin was irradiated with 0, 10, 30, or 50 Gy electron beams. Seventy days after radiation exposure, we observed no decrease in the overall survival of frogs exposed to different radiation doses (Figure 1B). Through hematoxylin and eosin (H&E) staining of skin tissues 58 days after irradiation, we detected a slight decrease in gland size in the dermis of skin exposed to 30 Gy and obvious rupture of the epidermis and tissue damage of the dermis in those exposed to 50 Gy (Figure 1C). Furthermore, to minimize the influence of individual differences and behavioral activity, we subsequently established the limb skin irradiation model, in which the right legs were irradiated and the left legs were set as controls (Figure 1D). Fifty Gy radiation caused swelling and other pathological changes in less than 30% of the frog skins (Figure 1E). Previously, we established reliable radiation‐induced skin injury models in mice, rats, mini‐pigs, and monkeys, in which mammals irradiated with 30 or 45 Gy electron beams exhibited obvious skin ulcers and even severe injury.^[^ ^25^, ^26^ ^]^ Therefore, compared to common model animals, frogs, due to the properties of their skin, indeed exhibited resistance to ionizing radiation.

### 2.2 Identification of Radiation‐Induced Frog Skin Peptides (RIFSPs) as Mitigators of Radiation‐Induced Skin Injury Through Multi‐Omic Analysis

Next, to understand the changes in frog skins caused by irradiation, we adopted ultraviolet‐visible absorption spectrometry (UV‐VIS) to analyze the crude extracts of frog skin tissues. 72 h after 30 Gy irradiation, the peak density in skin extracts was much higher than that in non‐irradiated or 24 h post‐irradiated tissues (Figure 1F,G), indicating altered components. To elucidate the exact change, we further monitored the gene profiles of irradiated skin tissues through RNA‐seq (Figure 1F). A total of 1411 differentially expressed genes were identified (with the threshold set for the fold change ≥ 2.0 and *P* value < 0.05), of which 536 genes were upregulated (GSE113944; Figure 1H,I). Then, using GO analysis, we found that the upregulated genes were enriched in peptidase inhibitory activity, peptidase regulator activity, endopeptidase inhibitory activity, and endopeptidase regulator activity (Figure 1J), while the downregulated genes mainly involved in peptide cross‐linking, ribosome biogenesis, cell redox and actin homeostasis (Figure S1A, Supporting Information). Besides, results from KEGG analysis revealed that the downregulated genes were mainly related to ribosomes (Figure 1K), whereas the upregulated genes were enriched in the oxidative phosphorylation pathway (Figure S1B, Supporting Information). These results suggested the suppression of protein synthesis and mobilization of peptidases in irradiated frog skin tissues and we therefore speculated that irradiation altered the levels of several peptidases involved in proteolysis in the skin tissues of frogs, which may be responsible for the protective effect against radiation‐induced skin injury.

To verify this hypothesis, we subsequently used polypeptide‐omics to examine the peptide profiles in irradiated frog skin tissues (Figure 1F). The results revealed that irradiation caused significant changes in the peptides of the frog skin tissues (Figure 1L,M). Setting a fold‐change greater and less than 1.5 as the threshold, we identified a total of 26 peptides that were upregulated and 34 peptides that were downregulated in response to 30 Gy irradiation (Table S1, Supporting Information). Furthermore, through integrative analysis of data from RNA‐Seq and polypeptide omics, the upregulated peptides were regarded as fragments mainly from histones and ribosomes. We named these radiation‐induced frog skin peptides radiation‐induced frog skin peptides (RIFSPs) and found enhanced proliferation and migration ability in irradiated human skin cells administered upregulated RIFSPs, some of which also exerted antioxidant properties (Figure S1C–E, Supporting Information). Moreover, in comprehensive consideration of the ability to improve proliferation and migration and the anti‐oxidant capacity (Figure S1C–E, Supporting Information), we selected RIFSP‐2/5/9/10 to investigate their protective effects in vivo. After the generation of a combined radiogenic injury model through the combination of total body radiation (TBI) with 4.0 Gy dose of X‐ray irradiation and mechanical trauma on the buttocks of rats, RIFSP‐2/5/9/10 (15 µm in 20 µL) or an equivalent volume of PBS was administered daily to the superficial injury site, and the wound area was measured with Vernier calipers every other day until healed. The results of wound area curves showed acceleration of the healing process for rats treated with the four tested RIFSPs (Figure 1N,O). Then the potential synergy among these peptides was further investigated and results from the CCK‐8 and LDH assays failed to support the benefits of joint administration of RIFSPs (Figure S1F, Supporting Information). Hence, these peptides, with elevated levels, from histones or ribosomes, may facilitate the radiation resistance of frog skin tissues and exhibited radiation mitigation ability in animal models.

### 2.3 Macropinocytosis Mediates the Intracellular Uptake of RIFSP‐2 in HaCaT Cells

**Figure 2:**
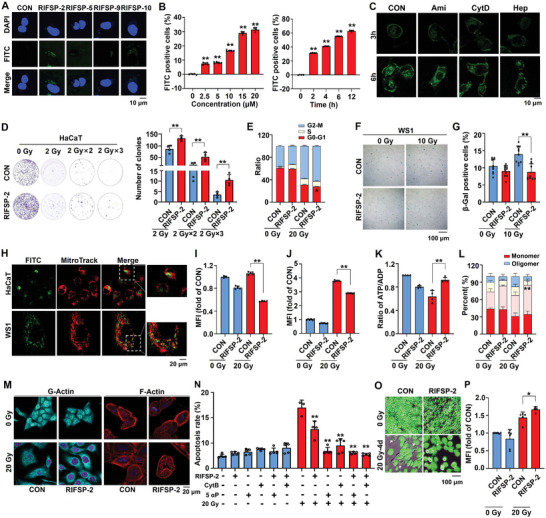
Macropinocytosis‐mediated uptake of radiation‐induced frog skin peptide 2 (RIFSP‐2) protects skin cells against radiation‐induced injury. A) Administration of FITC‐labeled RIFSPs to HaCaT cells and identification of the membrane impermeability of RIFSP‐2 through confocal microscopy. B) Time‐ and dose‐dependent uptake of FITC‐RIFSP‐2 by HaCaT cells detected through flow cytometry analysis. C) Influence of heparin, amiloride, and cytochalasin D on the uptake of FITC‐RIFSP‐2 by HaCaT cells through confocal microscopy detection. The effect of RIFSP‐2 on D) cell proliferation, E) cell cycle, and F,G) senescence, as detected by colony formation assay, propidium iodide (PI) staining and SA‐β‐Gal staining analysis, respectively. H) Colocalization analysis showing FITC‐RIFSP‐2 (green) and Mito‐Tracker (red) in irradiated HaCaT and WS1 cells. I–L) Influence of RIFSP‐2 on cellular ROS accumulation, mitochondrial ROS accumulation, energy production, and mitochondrial membrane potential in irradiated WS1 cells through DCFH‐DA staining, MitoSox probe staining, ADP probe staining and JC‐1staining, respectively. M) Effect of RIFSP‐2 on the homeostasis of actin filaments in irradiated skin cells, as detected by rhodamine phalloidin and G‐actin staining assays. N) The actin polymerization inhibitor, cytochalasin B (CytB, 20µm, 2h), and the actin depolymerization agonist, 5a‐Pregnane‐3,20‐dione (5αP, 100nm, 24h) were administrated before RIFSP‐2 treatment. Investigation of cell death in irradiated skin cells was adopted with AV/PI staining kits. O) and P) Calcein AM release assay for understanding the influence of RIFSP‐2 on lytic cell death in irradiated HaCaT cells. *P < 0.05 and **P < 0.01, compared with the control group.

Distinct from previous reports, the RIFSPs were obtained from skin tissue crude extracts but not from skin secretions. In addition, the administration of exogenous RIFSPs protected cultured skin cells, which hints that some RIFSPs may exert membrane permeability. Therefore, we synthesized FITC‐labeled RIFSPs‐2/5/9/10 and evaluated their possible intracellular uptake by skin cells. After incubation of 15 µm FITC‐RIFSPs with skin cells for 24 h, we detected an obvious intracellular FITC signal in RIFSP‐2‐treated cells but not in cells treated with the other RIFSPs (**Figure** **2**A; Figure S2A, Supporting Information). Moreover, uptake of RIFSP‐2 in HaCaT cells occurred in a time‐ and dose‐dependent manner (Figure 2B), while WS1 cells exhibited better permeability of RIFSP‐2, of which the FITC signal could be detected as soon as one hour after administration (Figure S2B, Supporting Information). Glycosaminoglycans have been reported to affect the efficiency of cellular uptake of a wide range of cell penetrating peptides,^[^ ^27^ ^]^ and we therefore used the competitive inhibitor heparin to investigate whether internalization of FITC‐RIFSP‐2 was dependent on recognition by glycosaminoglycans on the cell surface. The confocal microscopy and flow cytometry analysis results revealed that the addition of heparin substantially decreased the intracellular intensity of FITC (0.85‐fold for HaCaT cells and 0.86‐fold for WS1 cells treated with heparin) (Figure 2C; Figure S2C,D, Supporting Information), suggesting a vital role for cell surface glycosaminoglycans in the RIFSP‐2 internalization process.

After forming electrostatic interactions with the plasma membrane, peptides principally penetrate into cells via macropinocytosis.^[^ ^28^ ^]^ Therefore, to verify whether internalization of RIFSP‐2 is regulated by macropinocytosis, we used amiloride (Ami, a specific inhibitor of the Na^+^/H^+^ exchange required for macropinocytosis) and cytochalasin D (CytD, an F‐actin elongation inhibitor) to treat skin cells. The results revealed that each of the inhibitors significantly inhibited RIFSP‐2 entry into the cells. The mean fluorescence intensity of FITC‐RIFSP‐2 in Ami‐ or CytD‐ treated HaCaT cells were 0.58 and 0.84, respectively, which were all significantly decreased compared with those of the positive control. However, pretreatment of Ami caused only ≈5% decrease of fluorescence intensity in WS1 cells, while WS1 cells swelled and subsequently died in 30 min after CytD administration (Figure S2C,D, Supporting Information). Altogether, these results indicated that RIFSP‐2 translocated into HaCaT cells in a glycosaminoglycan‐ and macropinocytosis‐dependent manner and functioned as a potential radiation mitigator.

### 2.4 RIFSP‐2 Maintains Cellular Homeostasis and Reduces Radiation‐Induced Pyroptosis

To investigate the protective effects of RIFSP‐2 on irradiated skin cells, we performed series in vitro studies. Results of the colony formation analysis showed that RIFSP‐2 significantly increased the colony formation ability of HaCaT cells exposed to 2 Gy irradiation and protected cell viability of HaCaT cells against fractionated radiation (Figure 2D). Then, the results from cell cycle analysis revealed a greater proportion of irradiated HaCaT cells in the G2/M stage after treatment with RIFSP‐2, providing more time to repair the damaged DNA (Figure 2E). And data from SA‐β‐Gal staining after RIFSP‐2 treatment reflected that the cells exposed to radiation showed more intense positive staining, whereas notably less positive staining was observed in RIFSP‐2‐treated cells (Figure 2F,G). In light that mitochondria being regarded as multifaceted regulator of cell death,^[^ ^29^ ^]^ we further investigated the role of RIFSP‐2 in homeostasis of mitochondria in irradiated skin cells. Through colocalization analysis, we detected obvious merging of FITC‐labeled RIFSP‐2 and the MitoTracker signal (Figure 2H). With fluorescent probes that illustrate total level of cellular ROS (DCFH‐DA, Figure 2I), mitochondrial ROS accumulation (MitoSox, Figure 2J) and energy production (ADP‐probe, Figure 2K), we also found that pretreatment with RIFSP‐2 dramatically promoted energy production ability and reduced the level of mitochondrial and total ROS accumulation in irradiated WS1 cells, while limited alterations of mitochondrial function were observed in irradiated HaCaT cells (Figure S2E, Supporting Information). Then, JC‐1‐based staining indicated that RIFSP‐2 retained mitochondrial membrane potential in response to irradiation, as evidenced by the increase in the proportion of JC‐1 oligomers in irradiated HaCaT (Figure 2L) and WS1 cells (Figure S2F, Supporting Information). In addition, supplementation with medium from WS1 cells cultured and pretreated with RIFSP‐2 dramatically restrained the decline in mitochondrial membrane potential in irradiated HaCaT cells and vice versa (Figure S2G–I, Supporting Information). These results suggested that RIFSP‐2 protected mitochondrial function in irradiated skin cells.

Since RIFSP‐2 promoted wound healing, we next investigated the role of RIFSP‐2 in the homeostasis of actin filaments in irradiated skin cells through rhodamine phalloidin and G‐actin staining assays. As shown in Figure 2M, radiation induced the polymerization of G‐actin and the formation of actin filaments, whereas pretreatment with RIFSP‐2 attenuated this process. Through Annexin V/propidine iodide staining, we found that the proportion of membrane injured cells (AV+/PI‐) among the irradiated WS1 cells treated with RIFSP‐2 dramatically decreased and the protective effects of RIFSP‐2 were abolished by cytochalasin B, an inhibitor of actin polymerization, or 5a‐Pregnane‐3,20‐dione, an agonist of actin depolymerization (Figure 2N). In addition, the calcein release was partially blocked in irradiated skin cells after treatment with RIFSP‐2 (Figure 2O,P). Furthermore, we investigated the influence of RIFSP‐2 on cell pyroptosis signaling in mouse primary skin cells, human keratinocytes, and skin fibroblasts. The results revealed a significant decrease in the phosphorylation of TBK1 and the cleavage of Caspase‐1 and/ or GSDMD in irradiated skin cells (Figure S3A–C, Supporting Information). Altogether, these studies revealed that RIFSP‐2 affected the homeostasis of mitochondrial and cytoskeleton, which attenuated radiation‐induced pyroptosis.

To eliminate the interference of p53 mutations (R282Q and H179Y in HaCaT cells), we further adopted series functional analysis for the role of RIFSP‐2 in the first to fourth passages of primary normal human epidermal keratinocytes (HEKs, Figure S4A, Supporting Information). Administration of RIFSP‐2 significantly improved mitochondrial membrane potential (Figure S4B, Supporting Information) and cellular content of neutral lipid droplets (Figure S4C, Supporting Information). Although results from CCK‐8 failed to support the promotion of cell viability of RIFSP‐2 in 24 h after 20 Gy irradiation (Figure S4B, Supporting Information), we detected significantly increased number of Edu positive cells in 72 h (Figure S4E, Supporting Information). Besides, pretreatment of RIFSP‐2 restrained LDH release in the same time (Figure S4F, Supporting Information), accompanied with a reduction of AV+/ PI+ proportion of irradiated HEKs (Figure S4G, Supporting Information). Finally, results from SA‐β‐Gal staining revealed that RIFSP‐2 significantly attenuated senescence of HEKs in a week after 20 Gy irradiation. Therefore, RIFSP‐2 protected not only immortalized human skin keretinocyte and fibroblast cells, but it also protected primary human epidermal keratinocytes from ionizing irradiation, regardless of p53 mutation.

### 2.5 RIFSP‐2 Restrained SCD1‐Mediated Biogenesis of MUFA in Irradiated Skin Cells

**Figure 3:**
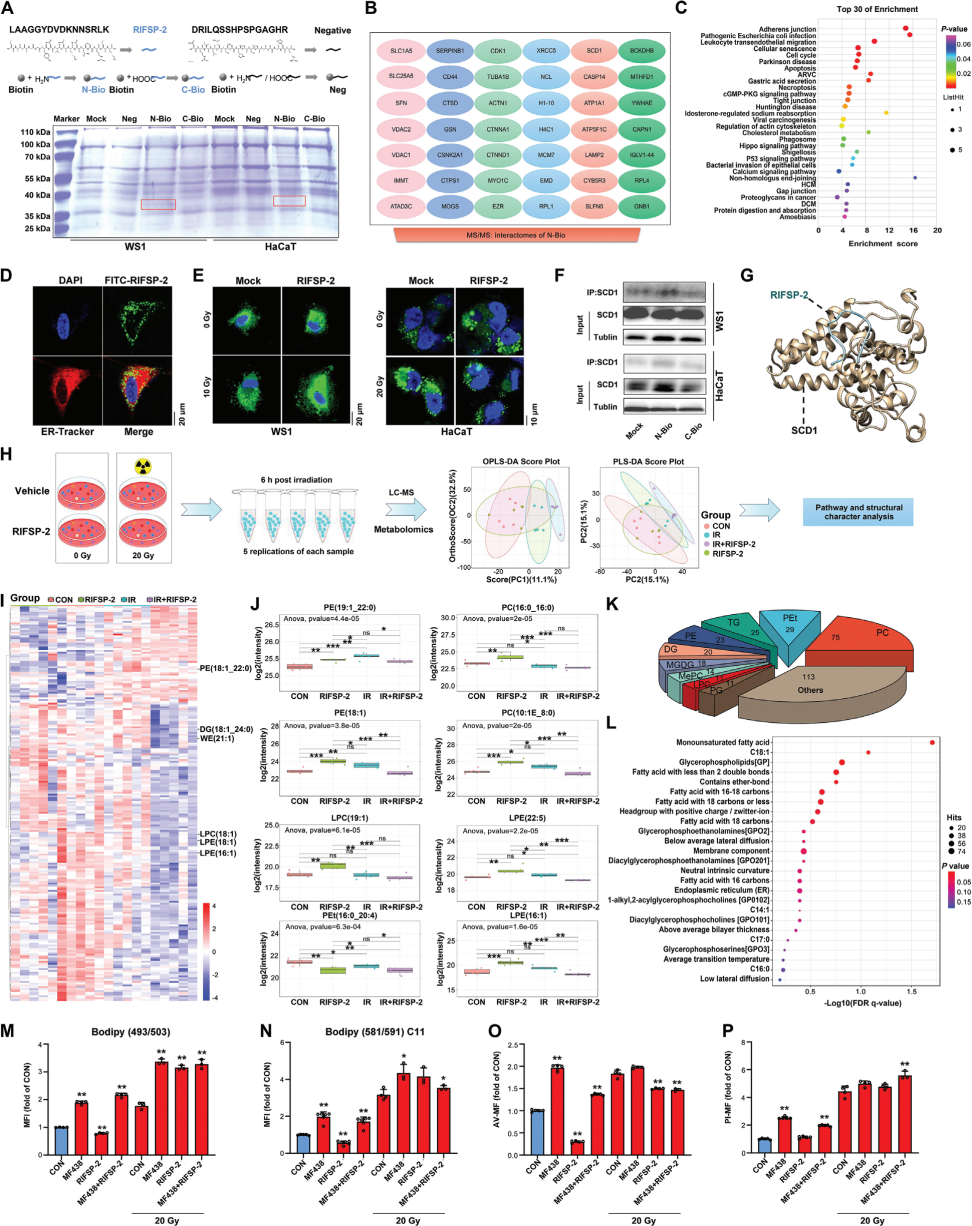
RIFSP‐2 restrained SCD1‐mediated biogenesis of MUFA in irradiated skin cells. A) Schematic showing the proteomic analysis performed to investigate the interactome of RIFSP‐2 in irradiated skin cells, as determined by liquid chromatography with tandem mass spectrometry (LC–MS/MS), based on the Avidin‐Biotin‐RIFSP‐2 system. B) Bradford staining of proteins pulled down with Biotin‐RIFSP‐2 and Venn diagram showing common and specific interactomes detected by LC–MS/MS and representative proteins. C) The enriched functions of RIFSP‐2 interactomes in irradiated skin cells based on Gene Ontology (GO) molecular function analysis. D) Colocalization analysis showing FITC‐RIFSP‐2 (green) and ER‐Tracker (red) in irradiated HaCaT cells. E) Changes in neutral lipid droplet formation in irradiated skin cells treated with RIFSP‐2 detected via BODIPY 493/503 probe staining. F) Verification of the interaction between RIFSP‐2 and SCD1 through immunoprecipitation (IP) analysis. G) Molecular simulation of the interaction between RIFSP‐2 and SCD1. H) Schematic showing the lipidomic analysis performed to investigate the alteration of lipid metabolism in irradiated HaCaT cells pretreated with or without RIFSP‐2. I) A heatmap revealed influence of ionizing radiation and RIFSP‐2 on lipid metabolite profiles in HaCaT cells. J) Boxplot depicting the lipids with the most significant differences in irradiated HaCaT cells pretreated with or without RIFSP‐2. K) Pie chart showing the distinct lipid metabolite profiles in irradiated HaCaT cells pretreated with or without RIFSP‐2. L) Functional enrichment analysis of the different metabolites in irradiated cells treated with or without RIFSP‐2. M–P) Influence of the SCD1 inhibitor MF438 on the neutral lipid droplet formation, ferroptosis, and apoptosis of irradiated skin cells pretreated with RIFSP‐2, as detected by BODIPY 493/503 probe staining, BODIPY 581/591 C11 probe staining and AV/PI staining. *P < 0.05 and **P < 0.01, compared with the control group.

To investigate the potential direct targets of RIFSP‐2 in irradiated skin cells, we adopted a proteomic approach to investigate its interactome based on the streptavidin‐biotin system (**Figure** **3**A). Forty‐two unique proteins that interacted with RIFSP‐2 after irradiation were identified, including β‐actin (ACTB), inner membrane mitochondrial protein (IMMT), stearyl‐CoA desaturase 1 (SCD1), and so on (Figure 3B). Then, the KEGG analysis of those specific interactomes revealed that RIFSP‐2 affected processes related to DNA damage repair, cell cycle, senescence, necroptosis, Hippo, and P53 signaling (Figure 3C), which partially accounts for its property of radiation mitigator. Most recently, through integrative multi‐omic analysis, we reported that ionizing radiation induced the upregulation of SCD1 and subsequently increased the biogenesis of stearic acid and oleic acid in irradiated mouse skin tissues.^[^ ^30^ ^]^ Therefore, we further validated the interaction between SCD1 and RIFSP‐2. With specific probes for the endoplasmic reticulum (ER), we visualized localization of FITC‐RIFSP‐2 in ER (Figure 3D). Next, the results from BODIPY 493/503 probe staining revealed significant alteration of neutral lipid droplets in irradiated skin cells by RIFSP‐2 (Figure 3E). Then we confirmed the interaction between N‐terminal biotin‐labeled RIFSP‐2 and SCD1 through IP analysis, and molecular docking also reflected the potent interaction between RIFSP‐2 and SCD1 (Figure 3F,G). Thus, SCD1 sounds to be a direct target of RIFSP‐2 in irradiated skin cells.

Since SCD1 plays a vital role in triglyceride biosynthesis, restraint of SCD1 by RIFSP‐2 may modulate the homeostasis of lipid metabolism in response to radiation. To verify this hypothesis, we performed lipidomic analysis of irradiated HaCaT cells pretreated with or without RIFSP‐2 (Figure 3H). The results revealed significant difference in the lipid metabolites of different groups (Figure 3I,J), among which PE (18:1_22:0) and Pet (16:0_20:4) were identified as common differentially expressed metabolites, with VIP > 1.0 and *p*<0.05 as the cutoff criteria (Table S2, Supporting Information). In addition, 54 metabolites were upregulated and 275 metabolites were downregulated in irradiated cells pretreated with RIFSP‐2 compared with irradiated cells (Figure 3K; Table S3, Supporting Information). Then, four communities were classified based on the association network of the different metabolites in irradiated cells treated with or without RIFSP‐2 (Figure S5A, Supporting Information) and functional enrichment analysis of class I community revealed that RIFSP‐2 treatment significantly affected MUFA biogenesis (Figure 3L). Functionally, the results from BODIPY 493/503 probe staining showed that pretreatment with both RIFSP‐2 and MF‐438, an inhibitor of SCD1, effectively promoted the formation of neutral lipid droplets in skin cells (Figure 3M). Moreover, RIFSP‐2 significantly decreased the intensity of ferroptosis and AV staining, whereas pretreatment with MF‐438 diminished these effects in nonirradiated cells (Figure 3N–P). Altogether, these results indicated that RIFSP‐2 impaired SCD1‐mediated biogenesis of MUFAs, which contributes to protection against oxidative membrane injury in irradiated skin cells.

### 2.6 RIFSP‐2 Impaired STING‐Mediated Inflammation Response by Disrupting the SCD1‐MUFA Axis in Irradiated Skin Cells

**Figure 4:**
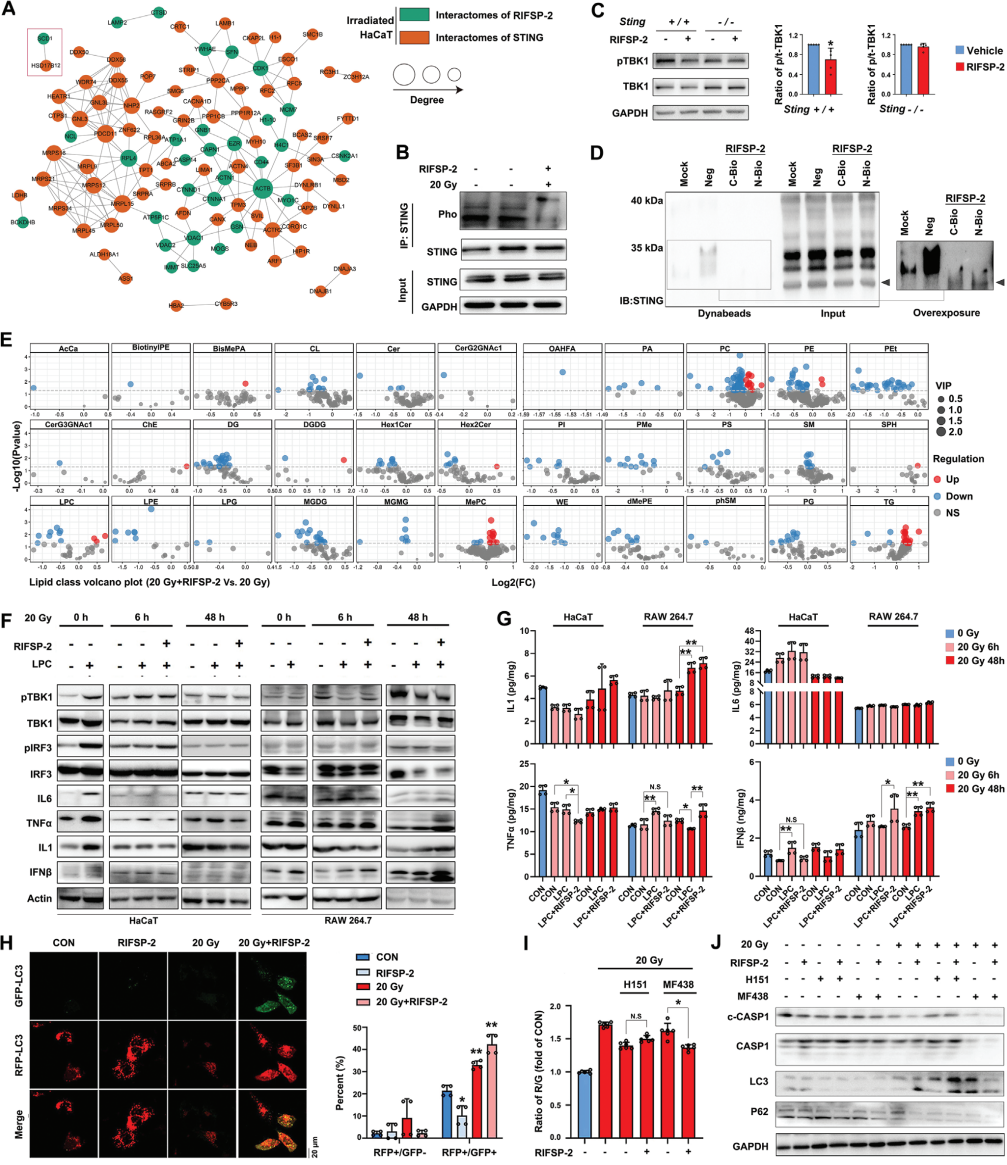
MUFAs facilitate ionizing radiation‐induced STING activation, which is disrupted by RIFSP‐2. A) Bioinformatic analysis of the connection between specific binding proteins of RIFSP‐2 (green nodes) and STING (red nodes) in irradiated skin cells. B) Detection of the phosphorylation of STING in RIFSP‐2‐treated and irradiated HaCaT cells through IP analysis. C) Detection of the phosphorylation of TBK1 in RIFSP‐2‐treated primary skin cells from *STING* wild type mice and depletion ones through Western blotting analysis. D) Investigation of the interaction between STING and RIFSP‐2 in irradiated HaCaT cells based on the established Avidin‐Biotin‐RIFSP‐2 system. E) Volcano plots of differences between lipid class in irradiated HaCaT cells pretreated with RIFSP‐2 or not. F) Influence of LPC (18:1) and RIFSP‐2 in STING downstream signals in irradiated HaCaT cells through Western blotting analysis. G) Influence of LPC (18:1) and RIFSP‐2 in STING downstream inflammatory factors in irradiated HaCaT and RAW 264.7 cells through ELISA assays. H) Confocal microscopy and flow cytometry analysis showing RIFSP‐2‐treated cells infected with mRFP‐GFP‐LC3; GFP+/RFP+ cells represent autophagosomes, and GFP+/RFP‐ cells represent autolysosomes. I) Flow cytometry analysis of mRFP‐GFP‐LC3‐infected HaCaT cells pretreated with 15 µm RIFSP‐2 in combination with H151 or MF438 in response to radiation. J) Alteration of biomarkers for autophagy and pyroptosis in irradiated HaCaT cells pretreated with 15 µm RIFSP‐2 in combination with H151 or MF438, detected by Western blotting. *P < 0.05 and **P < 0.01, compared with the control group. N.S, non‐significant.

Considering the localization of RIFSP‐2 in ER, we then investigated the potential connection between RIFSP‐2 and STING. First, we adopted a proteomic analysis to investigated the interactomes of STING in HaCaT cells, and identified 136 specific proteins in response to radiation. Then, strong connections between specific interactomes for RIFSP‐2 and STING in irradiated skin cells were established in protein interactive analysis, with confidence score > 0.7 (high confidence) (**Figure** **4**A). To testify whether RIFSP‐2 influences radiation‐induced STING activation, we adopted IP analysis and found that RIFSP‐2 reduced STING phosphorylation in irradiated skin cells (Figure 4B). Besides, RIFSP‐2 was proved to significantly reduce TBK1 phosphorylation in primary skin cells from wild‐type mice but not the *Sting* depletion ones (Figure 4C). Administration of H151, an antagonist of STING phosphorylation, also suppressed downregulation of TBK1 phosphorylation in irradiated skin cells treated with RIFSP‐2 (Figure S5E, Supporting Information). Although, results from the surface plasmon resonance (SPR) assay showed a dissociation constant of 228.53 (Figure S5D, Supporting Information), we detected a minor and specific binding band of STING for biotin‐labled (Bio‐) RIFSP‐2 in irradiated HaCaT cells when overexposed (Figure 4D). These results highlighted the suppression of STING activation by RIFSP‐2 in irradiated skin cells.

Recently, accumulating evidence suggests the reciprocal modulation between STING and lipid metabolism.^[^ ^31^, ^32^ ^]^ Since RIFSP‐2 directly restrained SCD1 and modulated neutral lipid droplets, we further investigated whether alteration of lipid profiles by RIFSP‐2 interfered with STING activation in response to irradiation. Volcano plot of lipid class reflected that administration of RIFSP‐2 resulted in obvious change of cardiolipins (CL), diglyceride (DG), lyso‐phosphatidylcholine (LPC), lyso‐phosphatidylethanolamine (LPE), monogalactosyldiacylglycerols (MGDG), methylphosphocholine (MePC), phosphatidylcholines (PC), phosphatidylethanolamines (PE), phosphatidylethanol (PEt), phosphatidylmethanol (PMe) and triglyceride (TG) (Figure 4E). And results from the structural character analysis revealed the significantly negative coefficient between MUFA and RIFSP‐2 treatment in irradiated HaCaT cells (Figure S5B). Then, we selected LPC (18:1) as the candidate metabolite and the results revealed that supplementation with LPC (18:1) elevated the phosphorylation of both STING and its substrate TBK1 in a dose‐dependent manner (Figure S5C, Supporting Information). Furthermore, we investigated the influence of RIFSP‐2 on STING‐regulated inflammatory factors, including IFN‐β, TNF‐α, IL‐1, and IL‐6. Results from Western blotting analysis revealed that administration of LPC (18:1) aggravated the accumulation of TGF‐β, TNF‐α, IL‐1, and IL‐6 in keratinocytes but not macrophage cells (Figure 4F). In acute stage (6 h) after irradiation, RIFSP‐2 treatment reduced the level of IFN‐β and TNF‐α in keratinocytes exposed to LPC (Figure 4F). Nevertheless, significant increase of IFN‐β production in acute stage and TNF‐α production in relative late stage (48 h) in macrophage cells were detected through both Western blotting and ELISA assays (Figure 4F,G). Therefore, RIFSP‐2 indirectly inhibited STING‐mediated inflammation response partially in a SCD1‐MUFA‐related manner in irradiated skin cells.

### 2.7 RIFSP‐2 Promotes Ubiquitin Proteasome System‐Mediated Degradation of TBK1 and SCD1‐Regulated Autophagy

Significant decrease of TBK1 protein level was observed in RIFSP‐2 treated HaCaT cells but not WS1 cells in Figure S3A,B (Supporting Information). Here, we further explored whether and how RIFSP‐2 decreased the protein level of TBK1 in HaCaT cells. First, we detected the reduction of TBK1 protein levels by RIFSP‐2 treatment in a dose‐ and time‐dependent manner (Figure S5H, Supporting Information). Since the degradation of TBK1 protein was reported to be regulated by either ubiquitination mediated proteasome pathway^[^ ^33^ ^]^ or chaperone‐mediated autophagy signaling,^[^ ^34^ ^]^ we testified the influence of the protein synthesis inhibitor (CHX) and the proteasome inhibitor (MG132) in RIFSP‐2 treated HaCaT cells. The results revealed that the half‐life of TBK1 was around 12–24 h (Figure S5G, Supporting Information) and MG132 abrogated the reduction of TBK1 protein level in 24 h after RIFSP‐2 treatment (Figure S5I). Furthermore, we adopted IP analysis to investigate the change of ubiquitination of TBK1 in HaCaT cells treated with RIFSP‐2 and found that RIFSP‐2 triggered dramatic increase of TBK1 ubiquitination and overall ubiquitination in HaCaT cells (Figure S5J, Supporting Information). Then we analyzed the colocalization of TBK1 and lysosomes through confocal immunofluorescence microscopy assays. Although, administration of RIFSP‐2 induced a transient decrease of TBK1 fluorescence intensity (Figure S5K,L, Supporting Information), the co‐localization assays didn't support the increase of TBK1 location in lysosomes in RIFSP‐2 treated HaCaT cells (Figure S5M, Supporting Information). Then the lysosomal inhibitor (chloroquine, CQ) was used to ensure the role of lysosome in RIFSP‐2 mediated degradation of TBK1 and the results revealed little influence of lysosomes in TBK1 degradation caused by RIFSP‐2 (Figure S5N, Supporting Information). Altogether, these results reflected that RIFSP‐2 promoted the ubiquitination‐mediated degradation of TBK1 in HaCaT cells, which may contribute to the prevent the radiation‐induced STING activation in acute (within 24 h) stage.

Previously, the induction of autophagy was demonstrated to be an evolutionarily conserved function of the cGAS/STING pathway,^[^ ^35^ ^]^ and recently, mounting evidence has indicated that autophagy and lipid metabolism are tightly interconnected.^[^ ^36^ ^]^ We therefore explored the influence of RIFSP‐2 on autophagy. The results from the mRFP‐GFP‐LC3 infection study revealed an increased number of GFP+ autophagosomes in RIFSP‐2‐treated irradiated HaCaT cells (Figure 4H) and an elevated mean fluorescence intensity of both RFP and GFP in irradiated WS1 cells (Figure S3E, Supporting Information). Moreover, adjuvant administration of RIFSP‐2 failed to induce autophagy in irradiated cells pretreated with MF438 but not H151, as evidenced by the decrease in the ratio of R/G fluorescence intensity (Figure 4I). In addition, pretreatment with MF438 but not H151 dramatically limited the increase in LC3 II levels induced by RIFSP‐2 treatment (Figure 4J). Thus, RIFSP‐2 promoted radiation‐induced autophagy in an SCD1‐dependent manner, where STING translocation is unnecessary.

### 2.8 RIFSP‐2 Protects Against Radiation‐Induced Skin Injury in a STING‐Related manner In Vivo Without Toxicity

**Figure 5:**
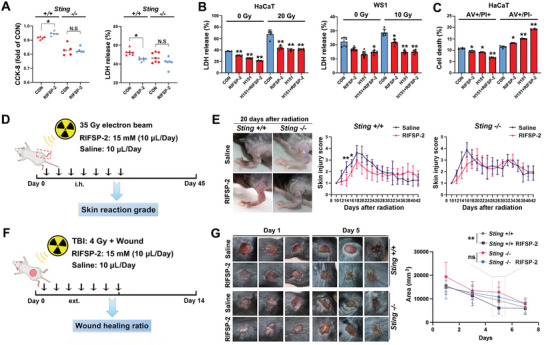
RIFSP‐2 mitigates radiation injury in a STING‐related manner. A) Cell proliferation and death assays for influence of RIFSP‐2 in primary skin cells from STING‐depleted and wild‐type mice detected by CCK‐8 and LDH analysis. B) LDH analysis of irradiated and nonirradiated skin cells pretreated with 15 µm RIFSP‐2 in combination with H151 or not. C) AV/PI and JC‐1 staining of irradiated HaCaT cells pretreated with 15 µM RIFSP‐2 in combination with or without H151. D,E) Schematic showing the methods of establishing radiogenic and combined skin injury mice models, administrated with RIFSP‐2. F) Pictures of typical radiation‐induced skin injury after irradiation and skin score grading of the radiation‐induced skin injury model mice administered 15 mm RIFSP‐2 or saline. G) Representative pictures of combined skin injury after irradiation and wound healing curve of the combined skin injury model mice administered 15 mm RIFSP‐2 or saline. *P < 0.05 and **P < 0.01, compared with the control group. N.S, non‐significant.

Next, we investigated whether suppression of the SCD1‐MUFA‐STING axis by RIFSP‐2 accounts for its protective effect against radiation injury in skin cells. Compared with primary skin cells from *Sting* depleted mice, administration of RIFSP‐2 increased the cell viability and decreased LDH release of those from wild‐type mice (**Figure** **5**A). Besides, *Sting* knockout dramatically limited the clonogenic formation ability after administration of RIFSP‐2 (Figure S4F, Supporting Information). Although both RIFSP‐2 and H151, inhibitors of STING translocation, attenuated LDH release in irradiated cells, pretreatment with H151 limited further protection against radiation‐induced lytic cell death (Figure 5B). However, the results from AV/PI staining revealed that the combination of RIFSP‐2 and H151 expanded the number of cells with membrane injury (AV+/PI‐) and further reduced the number of cells with nuclear damage (AV+/PI+) (Figure 5C). The JC‐1 staining results showed that RIFSP‐2 reversed the H151‐induced decline in mitochondrial membrane potential in irradiated skin cells (Figure S3D, Supporting Information). These results highlighted that RIFSP‐2 reduced radiation‐induced cell death partially in a STING‐dependent manner.

**Figure 6:**
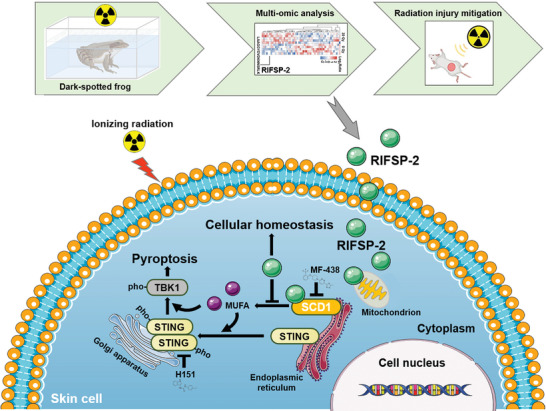
Schematic representation of RIFSP‐2 mitigating radiation‐induced skin injury. RIFSP‐2 from dark‐spotted frog is incorporated into skin cells and directly interacts with SCD1, which restrains radiation‐induced biogenesis of MUFA. Reduction of MUFA biogenesis limited phosphorylation of STING and subsequent activation of TBK1 and pyroptosis in irradiated skin cells. RIFSP‐2 maintains cellular homeostasis in a SCD1/STING related manner.

We next analyzed the involvement of STING in the radiation mitigation role of RIFSP‐2 in vivo using WT *Sting* (*Sting* ^+/+^), heterozygous (*Sting* ^+/−^), and deficient (*Sting* ^−/−^) mice (Figure S3F, Supporting Information). The hind limb skin tissues of the mice were irradiated with a single dose of 35 Gy electron beam irradiation to generate radiation‐induced skin injury, and then the skin tissue injuries were graded on a scale of 1 (no damage) to 5 (severe damage) as previously described (Figure 5D). Obvious mouse skin cutaneous injury began 10 days after 35 Gy irradiation and reached a maximum at 18 days, after which the wounds began to heal. Compared to the wild‐type mice, the *Sting* ^−/−^ mice benefitted less from RIFSP‐2 treatment (Figure 5E). Then, a combined injury model was established by treating mice with the combination of total body irradiation (TBI) with 4 Gy X‐ray irradiation and wound generation on the back (Figure 5F). As expected, RIFSP‐2 treatment promoted healing of the combined injury in wild‐type mice but scarcely affected healing in *Sting*‐deficient mice (Figure 5G). Altogether, these results showed that RIFSP‐2 protected against radiation‐induced skin injury through inhibition of the SCD1/STING pathway (**Figure** **6**).

In terms of potential toxicity, RIFSP‐2 was predicted to be a nontoxic peptide through ToxinPred software (https://webs.iiitd.edu.in/raghava/toxinpred/index.html). Moreover, to ensure the safety of RIFSP‐2 in vivo, we established two kinds of mouse models and administered RIFSP‐2 through external use or subcutaneous injection. And the histological examination of the skin tissues revealed no significant differences among irradiated skin tissues treated with RIFSP‐2 (Figure S6, Supporting Information). These results indicated that RIFSP‐2 treatment did not cause obvious adverse effects at the indicated dosage and may be effective in clinical settings.

## 3 Discussion

Distinct from small chemical molecules or antibodies, peptide therapeutics have become a rapidly developing strategy to target or inhibit protein–protein interactions as a result of their distinct biochemical and therapeutic properties.^[^ ^37^ ^]^ However, due to the intrinsic drawbacks of peptide drugs, such as in vivo instability and membrane impermeability, only a few peptide candidates have been clinically administered worldwide.^[^ ^38^ ^]^ For the treatment of skin injury, topical application eliminates the influence of plasma clearance, and in the past few decades, numerous peptides have been identified in the skin secretions from frogs that have antimicrobial, antioxidant, or anti‐inflammatory properties, making these secretory peptides potential candidates for promoting skin wound healing.^[^ ^39^, ^40^ ^]^ Moreover, recently exposure to high levels of ionizing radiation was reported to result in darker coloration in Chornobyl tree frogs.^[^ ^41^ ^]^ Hence, in addition to secretions, alterations in endogenous substances in irradiated frog skin tissues may also function as buffering mechanisms against ionizing radiation. In the current study, a novel endogenous peptide (RIFSP‐2) was identified in frog skin crude extracts but not collected secretions. RIFSP‐2 expression is induced by ionizing irradiation and derived from proteolysis of histones and serves as a promising radiation mitigator. Moreover, by investigating whether the histone sequence was evolutionally conserved, we observed that no species shared the same sequence of “LAAGGYDVDKNNSRLK” with amphibians (Figure S7A, Supporting Information). The potential cleavage sites for this sequence were further predicted through *PROSPER*,^[^ ^42^ ^]^ of which Elastase‐2, CTSG, MMP3, or MMP9 may execute protease cleavage^[^ ^43^, ^44^ ^]^(Figure S7B, Supporting Information). Thus, histone proteolysis may be a specific strategy for frogs to combat widespread radiation in the environment.

Through integrative multi‐omic analysis, we recently reported the alteration of fatty acid metabolism in the early response to ionizing radiation. In irradiated mouse skin tissues, we found that the expression of SCD1 and several genes related to MUFA metabolism were significantly upregulated.^[^ ^30^ ^]^ In this study, the results from the proteomics and metabolism analysis regarded SCD1 as a direct target of RIFSP‐2, which also dramatically impaired the process of MUFA biogenesis in the acute response to radiation. Through in vitro and in vivo functional exploration, we observed that RIFSP‐2 significantly reduced the release of LDH and subsequent cell death in irradiated cells. Previously, deletion of the gene encoding *SCD1* was reported to trigger cGAS‐STING‐mediated IFN‐I‐related immune responses in virus‐infected T cells,^[^ ^36^, ^45^, ^46^ ^]^ which suggests that SCD1 suppresses STING activation. Although STING was not the direct target of RIFSP‐2, we found a significant reduction in STING phosphorylation in irradiated cells treated with the SCD1 inhibitor MF438 or RIFSP‐2 through IP analysis. Furthermore, RIFSP‐2 treatment only protected wild‐type mice but not *Sting* depletion (*Sting* ^−/−^) mice from radiation‐induced skin injury. Therefore, suppression of SCD1 by RIFSP‐2 resulted in the inhibition of STING in irradiated skin tissues, and variations in STING activation may originate from the distinct lipid metabolism products in different cells. In addition, results from this study revealed that RIFSP‐2 promoted the ubiquitination‐mediated degradation of TBK1 in non‐irradiated HaCaT cells, which means the inhibition of the STING‐mediated basal IFN‐response state by RIFSP‐2.

Notably, apart from SCD1, there are other 41 distinct binding proteins of RIFSP‐2 were identified. The exact role of the other interactomes of RIFSP‐2 in its protection effect remains to be illustrated. Although the well membrane impermeability was identified, the half‐life of cellular RIFSP‐2 was not figured out. And whether the PEGylation or other modification could extend its half‐life and protective effects^[^ ^38^ ^]^? Besides, ultraviolet (UV) absorption by the skin triggers not only local cutaneous injury but also secondary systemic changes, like the neuroendocrine homeostasis.^[^ ^47^ ^]^ Peptides secretion has been regarded as a defense against UV in amphibians.^[^ ^13^ ^]^ And the pharmacological inhibition of STING was recently reported to restrain UV‐induced skin injury.^[^ ^48^ ^]^ Therefore, it's worthwhile to investigate whether the RIFSP‐2, targeting SCD1/ STING signal also serves for sunburn.

In summary, this study illustrates that SCD1‐mediated biogenesis of MUFAs aggravate STING‐mediated cell injury. As a novel radiation mitigator and inhibitor of SCD1, RIFSP‐2 exhibits suitable membrane permeability and may also be a promising candidate for radiation‐induced skin injury (Figure 6). This study hints that rich pharmacological resources innately exist in organisms, which will allow for discovering how to battling currently uncurable diseases and is likely to be key for future therapeutics.

## 4 Experimental Section

### Cell Culture and Irradiation

The HaCaT cell line (human keratinocytes) was obtained from the German Cancer Research Center (Heidelberg, Germany). The WS1 cell line (human skin fibroblasts) was purchased from ATCC. The normal human epidermal keratinocytes (HEKs) were purchased from (IMMOCELL, China). RAW 264.7 cell line was purchased from the Shanghai Cell Bank of the Chinese Academy of Sciences. The D‐KSFM medium was used for HEKs culture and bought from IMMOCELL (Xiamen, China). Other cells were maintained in Dulbecco's modified Eagle's medium (DMEM) and supplemented with 10% fetal bovine serum (FBS; Gibco). The cells were grown at 37°C in incubators with 5% CO_2_ and exposed to different dosages of ionizing radiation from an X‐ray linear accelerator (KUBTEC XCELL 320, Milford, CT) at a fixed dose rate of 1.7 Gy min^−1^, as previously reported.^[^ ^20^, ^25^ ^]^

### Frogs and Treatment

Artificially bred and farmed adult dark‐spotted frogs (*Pelophylax nigromaculatus*) weighing 20–25 g were obtained from a breeding base in Mianyang, China. After collection, frogs were provided with free access to water and housed under a 12‐h light/dark cycle. For irradiation, the back and legs of frogs were fixed in a plastic installation with no anesthesia, and then different doses of irradiation were produced by the accelerator (Clinac 2100EX; Varian Medical Systems, Palo Alto, CA) at a dose rate of 1000 cGy min^−1^ by a 6‐MeV electron beam. The frog experiment protocols were approved by the Animal Experimentation Ethics Committee at Soochow University (Suzhou, China).

### Synthesis of RIFSPs

RIFSPs, FITC‐labeled peptides, and Biotin‐labeled peptides were synthesized by Sangon Biotech Co., Ltd. (Shanghai, China), with a confirmed purity higher than 95% by HPLC and LCMS spectrometry.

### Mice and Treatment

*TMEM173*‐KO (*Sting* ^−/−^) and heterozygous (*Sting^+/−^*) mice were established by GemPharmatech, with exon 1 to exon 8 of the *Tmem173* transcript (ENSMUST00000115 728.4) excised using CRISPR/Cas9 technology (stock number T012747; Nanjing, China). Male C57BL/6 mice that were 6–8 weeks old and weighed 22–25 g and Sprague–Dawley (SD) rats 6–8 weeks old and weighing 200–250 g were purchased from GemPharmatech (Chengdu, China). The mice and rats were housed under a 12‐h light/dark cycle and had free access to food and water. To establish the combination skin models, mice and rats were anesthetized with an intraperitoneal injection of pentobarbital sodium (1%, 30 mg kg^−1^) and then lacerated on the back (diameter of 8 mm) immediately after 4 Gy total body irradiation (TBI) at a dose rate of 200 cGy min^−1^ using a 6‐MeV X‐ray (*n* = 6) with the accelerator (Clinac 2100EX; Varian Medical Systems, Palo Alto, CA) as reported previously.^[^ ^42^ ^]^ The mouse experiment protocols were approved by the Animal Experimentation Ethics Committee at Soochow University (Suzhou, China).

### Multi‐Omic Studies

To ensure the credibility of the results, omics studies were performed by different high‐quality biotechnology companies. RNA‐Seq of irradiated frog skin tissues was performed by OE Biotech (Shanghai, China). The polypeptide and interactome analyses were performed by PTM BioLab, Inc. (Hangzhou, China). Lipidomic analysis was performed by PANOMIX Biomedical Tech Co., Ltd. (Suzhou, China) as previously reported.^[^ ^25^ ^]^ The experimental details are included in the Supplementary Material.

### Immunoprecipitation (IP) Study

Cells were subjected to indicated treatments. Then, the cells were washed with prechilled phosphate‐buffered saline (PBS) and lysed with RIPA buffer containing protease inhibitor compounds and PMSF (1%). Cell lysates (500 µg) were subjected to immunoprecipitation with 3 µg STING antibodies for IP using Protein G Magnetic Beads (MedChemExpress). The protein level was detected by the indicated antibodies with Western blotting analysis.

### Biotin Streptavidin System for Interactome Study

The biotin‐labeled peptides were produced by Sangon Biotech Co., Ltd. (Shanghai, China), and 15 µm biotin‐labeled peptides were given to cultured cells 24 h before irradiation. Then, the irradiated cells were washed with prechilled phosphate‐buffered saline (PBS) and lysed with RIPA buffer containing protease inhibitor compounds and PMSF (1%). Five hundred micrograms of cell lysates were subjected to streptavidin magnetic beads for further interactome analysis and IP study.

### Surface Plasmon Resonance (SPR) Analysis

SPR‐based analysis (Anhui Gene Universal Biotech Co., Ltd., Chuzhou, China) was used to investigate the binding kinetics and affinity of RIFSP‐2 to STING. For the binding study with RIFSP‐2, purified human recombinant STING protein (Homo sapiens, Ensembl:ENSG00000184584; > 95%purity; MedChemExpress, Shanghai, China) was immobilized on a CM5 sensor chip using standard amine coupling in 10 mm HEPES (pH 7.0) to a level of ≈300 RU. Then, the surface was deactivated using 1.0 m G21ethanolamine‐HCl pH 8.5 and regenerated with 350 mm EDTA to remove any remaining unbound ligand. Then, RIFSP‐2 was dissolved to 5.68 mm with HEPS buffer and diluted to 1000 µm, 500 µm, 250 µm, 125 µm, 62.5 µm, 31.25 µm, and 0 nm. Finally, interaction studies between RIFSP‐2 and STING were performed at 25°C in HBS‐EP+ (10 mm HEPES, 150 mm NaCl, 3 mm EDTA, 0.05% surfactant P20; pH 7.4). Serial dilutions of RIFSP‐2 were injected at 30 µL min^−1^ using multiple cycle kinetics. NaOH (10 mm) was used to regenerate the surface. Apparent binding kinetics (KD, ka, and kd) were derived after fitting the experimental data to the 1:1 Langmuir binding model in Biacore T200 Evaluation Software 3.1.

### Statistical Analysis

An unpaired 2‐tailed Student's *t* test was used to compare the means of two groups. Multiple comparisons were performed with one‐way ANOVA with post hoc tests by Tukey's test. A *P* value less than 0.05 was considered statistically significant for all tests. Other materials and methods are detailed in the Supplementary Materials and Methods.

## Conflict of Interest

The authors declare no conflict of interest.

## Author Contributions

F.G., L.Z., and T.Y. contributed equally to this work. J.Z., J.C., and S.Z. performed conceptualization. L.Z., D.Y., and Z.H. performed methodology. F.G., Z.L., T.Y., F.J., P.Y., and J.C. performed investigation. T.Y., Z.Y., and B.S. performed visualization. Z.H., S.Z., and F.G. performed funding acquisition. S.Z. and J.C. performed project administration. S.Z. and J.Z. performed supervision. F.G. and T.Y. performed writing of original draft. S.Z. and J.Z. performed writing, reviewing & editing.

## Supporting information

Supporting Information

## Data Availability

The data that support the findings of this study are available from the corresponding author upon reasonable request.
