# Supplementary material for: A Frog Skin‐Derived Peptide Targeting SCD1 Exerts Radioprotective Effects Against Skin Injury by Inhibiting STING‐Mediated Inflammation
Source: Adv Sci (Weinh). 2024 Apr 6;11(25):2306253. doi: 10.1002/advs.202306253 (PMC11220654; doi:10.1002/advs.202306253)
Supplement: Supplementary file 1 — Supporting Information [file ADVS-11-2306253-s001.pdf]

ADVANCED SCIENCE
Open Access

Supporting Information

for *Adv. Sci.*, DOI 10.1002/advs.202306253

A Frog Skin-Derived Peptide Targeting SCD1 Exerts Radioprotective Effects Against Skin Injury by Inhibiting STING-Mediated Inflammation

Fenghao Geng, Li Zhong, Tingyi Yang, Jianhui Chen, Ping Yang, Fengdi Jiang, Tao Yan, Bin Song, Zuxiang Yu, Daojiang Yu, Jie Zhang, Jianping Cao* and Shuyu Zhang**

Supplementary materials and methods

1. Materials

1.1 Antibodies

Antibodies	Source	Identifier (Cat number)
Anti-GAPDH antibody	Abcam	ab9485
Anti-SCD1 antibody	Cell Signaling Technology	2794
Anti-STING antibody	Cell Signaling Technology	13674
Anti-P62 antibody	Cell Signaling Technology	39749
Anti-LC3B antibody	Abcam	ab192890
Anti-Caspase-1 antibody	Abcam	ab207802
Anti-Cleaved-Caspase 1 (Asp297) Antibody	Cell Signaling Technology	4199
Anti-GPX4 antibody	Cell Signaling Technology	52455
Anti-FACL4 antibody	Abcam	ab155282
Anti-Bax antibody	Cell Signaling Technology	2722
Anti-Bcl2 antibody	Cell Signaling Technology	15071
Anti-Caspase-8 antibody	Abcam	ab25901
Anti-Cleaved-Caspase 8 Antibody	Cell Signaling Technology	9496
Pyroptosis Antibody Sampler	Cell Signaling Technology	43811
Apoptosis/Necroptosis Antibody Sampler Kit	Cell Signaling Technology	92570
Goat anti-Rabbit IgG (H+L) Highly Cross-Adsorbed Secondary Antibody, Alexa Fluor™ Plus 555	Invitrogen	A32732
Goat anti-Mouse IgG (H+L) Highly Cross-Adsorbed Secondary Antibody, Alexa Fluor™ Plus 647	Invitrogen	A32728

1.2 Reagents and materials

Reagents	Source	Identifier
MF-438	MedChemExpress	HY-15822
H-151	MedChemExpress	HY-112693
Heparin	MedChemExpress	HY-17567
Amiloride	MedChemExpress	HY-B0285
Cytochalasin D	MedChemExpress	HY-N6682
LPC (18:1)	MedChemExpress	19420-56-5
Protein G magnetic beads	MedChemExpress	HY-K0204
Streptavidin magnetic beads	MedChemExpress	HY-K0208
BODIPY (493/503)	MedChemExpress	HY-W090090
BODIPY (581/591) C11	MedChemExpress	HY-D1301
DPPH	MedChemExpress	HY-112053
MitoSOX Red	MedChemExpress	HY-D1005
Enhanced mitochondrial membrane potential assay kit with JC-1	Beyotime Biotechnology	C2003S
LDH assay kit	Beyotime Biotechnology	C0016
CCK-8 assay kit	Beyotime Biotechnology	C0037
Calcein AM Cell Viability Assay Kit	Beyotime Biotechnology	C2013S
Apoptosis and Necrosis Assay Kit	Beyotime Biotechnology	C1056
Cell Cycle and Apoptosis Analysis Kit	Beyotime Biotechnology	C1052
Actin-Tracker Red-Rhodamine	Beyotime Biotechnology	C2207S
Mito-Tracker Red CMXRos	Beyotime Biotechnology	C1035
T-AOC Assay Kit	Beyotime Biotechnology	S0119
DAPI	Beyotime Biotechnology	C1002
Reactive Oxygen Species Assay Kit	Beyotime Biotechnology	S0033S
ER-Tracker™ Red (BODIPY™ TR Glibenclamide)	Thermo Fisher Scientific Inc	E34250

MitoTracker™ Red FM	Thermo Fisher Scientific Inc	M22425
G-Actin staining	ShangHai YiJi Industrial Co., Ltd	
mRFP-GFP-LC3	HanBIO Tech	HB-AP210 000
Chloroquine	Selleck	S6999
Lyso-Tracker Red	Beyotime Biotechnology	C1046

2. Methods

2.1 RNA-Seq analysis

Skin tissues from the back of frogs 48 h after 30 or 0 Gy irradiation were collected for RNA-Seq (GSE113944). RNA extraction and microarray profiling were performed in the laboratory of Novogene Co., LTD (Beijing, China). Briefly, RNA quantification and qualification was checked using NanoPhotometer® spectrophotometer (IMPLEN, CA, USA) and Qubit® RNA Assay Kit in Qubit® 2.0 Flurometer (Life Technologies, CA, USA). And the RNA integrity was assessed using the RNA Nano 6000 Assay Kit of the Agilent Bioanalyzer 2100 system (Agilent Technologies, CA, USA). For library preparation, a total amount of 1.5 μg RNA per sample was used as input material for the RNA sample preparations. Sequencing libraries were generated using NEBNext® Ultra™ RNA Library Prep Kit for Illumina® (NEB, USA) following manufacturer's recommendations and index codes were added to attribute sequences to each sample. Then for clustering and sequencing, the clustering of the index-coded samples was performed on a cBot Cluster Generation System using TruSeq PE Cluster Kit v3-cBot-HS (Illumia) according to the

manufacturer’s instructions. After cluster generation, the library preparations were sequenced on an Illumina Hiseq platform and paired-end reads were generated. And finally, informatic analysis were adopted on demand.

2.2 Label free quantitative peptidome analysis

Skin tissues from the back of frogs 48 h after 30 or 0 Gy irradiation were collected and grinded by liquid nitrogen into cell powder and then transferred to a 5-mL centrifuge tube. After that, four volumes of lysis buffer (8 M urea, 1% Protease Inhibitor Cocktail) was added to the cell powder, followed by sonication three times on ice using a high intensity ultrasonic processor (Scientz). The remaining debris was removed by centrifugation at 12,000 g at 4 °C for 10 min. Finally, the supernatant was collected and the protein concentration was determined with BCA kit according to the manufacturer’s instructions. For digestion, the protein solution was reduced with 5 mM dithiothreitol for 30 min at 56 °C and alkylated with 11 mM iodoacetamide for 15 min at room temperature in darkness. The protein sample was then diluted by adding 100 mM NH4HCO3 to urea concentration less than 2M. Finally, trypsin was added at 1:50 trypsin-to-protein mass ratio for the first digestion overnight and 1:100 trypsin-to-protein mass ratio for a second 4 h-digestion. Then the tryptic peptides were fractionated into fractions by high pH reverse-phase HPLC using Agilent 300Extend C18 column (5 μm particles, 4.6 mm ID, 250 mm length). And for further LC-MS/MS analysis, the tryptic peptides were dissolved in 0.1% formic acid (solvent A), directly loaded onto a home-made reversed-phase analytical column (15-cm length, 75 μm i.d.). The gradient was comprised of an increase from 6% to 23% solvent B (0.1% formic acid in 98% acetonitrile) over 26 min, 23% to 35% in 8 min and climbing to 80% in 3 min then holding

at 80% for the last 3 min, all at a constant flow rate of 400 nl/min on an EASY-nLC 1000 UPLC system. The peptides were then subjected to NSI source followed by tandem mass spectrometry (MS/MS) in Orbitrap FusionTM TribridTM (Thermo) coupled online to the UPLC. And finally, the resulting MS/MS data were processed using Maxquant search engine (v.1.5.2.8). Tandem mass spectra were searched from reverse decoy database. Label-free quantification method was LFQ, FDR was adjusted to < 1% and minimum score for peptides was set > 40.

2.3 Proteomics analyses

STING was pulled down through the specific antibody (CST, #13647) from HaCaT cells 6 h after 20 or 0 Gy irradiation. Then the gel was sent to JINGJIE PTM BioLab (Hangzhou, China). Analysis of interactomes of STING was adopted according to the standard protocols.

2.4 Liquid chromatography–mass spectrometry (LC-MS)-based lipidomic analysis

Six hours after 20 Gy irradiation, HaCaT cells pretreated with RIFSP-2 (15 μM for 24 h) or not were collected and centrifuged for 5 min at 3000 × g at room temperature. Then, the cell samples were vortexed for 30 s at -20°C in 750 μL of mixed solvent (chloroform: methanol, 2:1, v/v) with 100 mg glass beads. Then, the sample was rapidly frozen in liquid nitrogen for 5 min and thawed at room temperature for further study. The separated organic layer was further vortexed with 500 μL of mixed solvent (chloroform: methanol, 2:1, v/v) twice. Finally, the samples were concentrated and dried under vacuum and dissolved in 200 μL isopropanol, and the supernatant was filtered through a 0.22 μm membrane to obtain the prepared samples for LC–MS, which were executed on a Thermo Scientific™ Q

Exactive Focus mass spectrometer (ThermoFisher Scientific) with spray voltages of 3.5 kV and -2.5 kV in the positive and negative modes, respectively. Sheath gas and auxiliary gas were set at 30 and 10 arbitrary units, respectively. The capillary temperature was 325°C. The Orbitrap analyzer scanned over a mass range of 150-2,000 m/z for a full scan at a mass resolution of 35,000. Data-dependent acquisition MS/MS experiments were performed using an HCD scan. The normalized collision energy was 30 eV. Dynamic exclusion was implemented to remove some unnecessary information from the MS/MS spectra. The levels of individual lipid metabolites in every sample preparation were normalized to the sum of the peak areas of the lipid metabolites within the sample lipid class. LC–MS data were processed with Simca-P 13.0 (Umetrics AB, Umea, Sweden), and this software was also used to perform principal component analysis (PCA) and partial least squares discriminant analysis (PLS-DA) to discriminate between the groups of the study.

2.5 Association analysis of databasefrom RNA-Seq and peptidome analysis of irradiated frog skin tissues.

The ORF sequences of obtained 56718 mRNAs from RNA-Seq was used to establish the peptide precursor protein sequence database (including 1412 significant differently expressed genes). And the association between the 1412 genes and 395 peptides identified in peptidome analysis were explored through MaxQuant software. And the 114 peptides were identified as fractions of proteins coded by 31 genes:

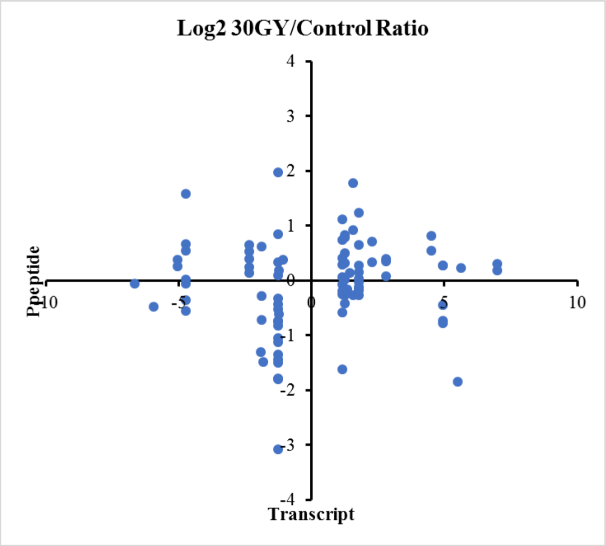

2.6 Protein-protein interaction analysis

The identified databases of specific binding proteins for RIFSP-2 and STING in irradiated skin cells were merged and the confidence score of interactions >0.7 were regarded as high confidence in alinement of STRING database. Finally, the visualization of this protein-protein interaction was realized through the Cytoscape software.

2.7 Assessment of cytokines by Elisa

HaCaT and RAW264.7 cells were cultivated in 6 cm plates at a density of 5×10^5 cells/mL with 2 mL medium per well and treated with LPC (18:1) plus RIFSP-2 for 24 h. 6 or 48 h after irradiation, cytokine concentrations in the cytoplasm were measured by ELISA according to the instructions provided by the manufacturer (NEOBIOSCIENCE). The absorbance was detected using a BioTek reader (Synergy HTX, Winooski, USA) at 450 nm. Logistic fitting-curve for the eight standard samples was employed for data processing.

2.8 H&E staining

Skin tissues were fixed in 10% neutral buffered formalin and embedded in paraffin. Three-micrometer paraffin sections were deparaffinized and heat-treated with citrate buffer at pH

6.0 for 7 min following an epitope retrieval protocol. The sections of frog skin were stained with H&E.

2.9 Clonogenic survival assay

A clonogenic survival assay of skin cells was performed as previously reported (1). HaCaT cells, WS1 cells and primary cells from wild-type (*Sting*$^{+/+}$) and *STING*-depleted (*Sting*$^{-/-}$) mouse skin tissues were pretreated with or without RIFSP-2 for 24 h and then exposed to single 2 Gy or fractionated 2 Gy X-ray radiation. After irradiation, cells were plated at a low density (1,000 cells per 6-cm plate), incubated for 10 days, fixed and stained with crystal violet. Colonies consisting of 50 or more cells were counted as a clone. The relative clonogenic survival fraction was calculated using ImageJ software as previously reported (1).

2.10 Cell viability assay

Cells were seeded in a 96-well plate at a density of 1 X 10^4 cells per well. Cells were then treated with the indicated concentration of RIFSPs and/or radiation. *In vitro* viability was measured using the Cell Counting Kit-8 (CCK-8) (Dojindo Laboratories, Kumamoto, Japan). Optical density was measured at 450 nm using a microplate reader (Biotek, Winooski, VT).

2.11 LDH release assay

Cell death was measured by the level of LDH released into the supernatant using an LDH cytotoxicity assay kit (Beyotime, Nantong, China) according to the manufacturer's instructions. The results are presented as the mean ± SEM of absorbance measured at 490 nm using a BioTek reader (Synergy HTX, Winooski, USA).

2.12 *In vitro* wound healing analysis

For the wound assay, 3 × 10^5 cells/well were plated into a 6-well plate and incubated to reach confluence. RIFSPs were pretreated 24 h before irradiation. After irradiation, the cells were scratched with a pipette tip, and then the detached cells were removed by washing with serum-free medium. The cells were photographed at 0 h and 24 h post wounding. The closure area of the wound was calculated as follows: migration area (%) = $(A_0 - A_{24})/A_0 \times 100$, where A0 represents the area of the initial wound area and A24 represents the remaining area of the wound at the metering point (2).

2.13 Immunofluorescence staining

HaCaT cells were fixed with 4% paraformaldehyde, washed with PBS, and permeabilized with 1% NP40 in PBS. Cells were blocked with blocking buffer (5% BSA) and incubated at 4°C with antibodies (1:200) against TBK1 overnight. FITC-conjugated goat anti-mouse/ rabbit (1:300) was added for 1 h at room temperature. The cells were observed under the Laser scanning confocal microscope (Olympus FV1000, Japan).

2.14 EdU incorporation assay

The proliferation of HEKs was determined by the uptake of EdU into DNA. Cells were pretreated with RIFSP-2 for 24 h and then exposed to 20 Gy irradiation. 72 hours after irradiation, cells in the logarithmic growth phase were trypsinized and measured by BeyoClick™ EdU-594 in vitro imaging kit (Beyotime Biotechnology, China). The cells were counter-stained with DAPI to visualize the nuclei and observed under a fluorescence microscope (Olympus, Tokyo, Japan).

Reference

1. Xue J, Zhu W, Song J, et al. (2018). Activation of PPARα by clofibrate sensitizes pancreatic cancer cells to radiation through the Wnt/β-catenin pathway. Oncogene. 37(7):953-962.

2. Gu Q, He Y, Ji J, Yao Y, Shen W, Luo J, Zhu W, Cao H, Geng Y, Xu J, Zhang S, Cao J, Ding WQ. Hypoxia-inducible factor 1α (HIF-1α) and reactive oxygen species (ROS) mediates radiation-induced invasiveness through the SDF-1α/CXCR4 pathway in non-small cell lung carcinoma cells. Oncotarget. 2015;6(13):10893-907.

Supplementary results

Table.S1 Details of Radiation-Induced Frog Skin Polypeptides（30Gy/Control）

		Peptide Quant		Upregulated						Transcript Quant	
**Number**	**Sequence**	**Length**	**Mass**	**Leading Razor Protein**	**Protein Description**	**Start Position**	**End Position**	**Ratio**	**P Value**	**Log2 Fold Change**	**Q value Storey**
1	TSYAQHQQVR	10	1216.595	g.58279	40S ribosomal protein S3a	176	185	1.716	5.26E-04	0.717	9.99E-01
2	LAAGGYDVDKNNSRLK	16	1719.890	g.34398	Histone H1A	81	96	1.538	2.44E-03	-1.155	9.99E-01
3	SGSGGGRISSGNFGSRSLQ	19	1809.872	g.65074	Keratin, type II cytoskeletal 75	72	90	1.758	2.44E-03	1.192	1.44E-05
4	EGTKAVTKYTSAK	13	1382.741	g.33075	Histone H2B 1.1	129	141	1.423	2.69E-03	0.620	9.99E-01
5	HFNAPSHVR	9	1063.531	g.32826	60S ribosomal protein L26	20	28	1.372	6.37E-03	0.555	5.71E-52
6	KAFAKRQQQLT	11	1317.752	g.69201	Myosin-11	809	819	1.488	8.24E-03	4.453	1.01E-139

7	AVSEGTKAV TKYTSAK	16	1639. 878	g.33075	Histone H2B 1.1	126	141	1.5 37	8.24 E-03	0.620	9.99E-01
8	NHYKVGDNA DSQIKL	15	1700. 848	g.67758	Histone H1.0	56	70	1.6 23	1.73 E-02	-0.033	9.99E-01
9	SGFARPGN WSSSSLSNV G	18	1808. 844	g.10449	Keratin, type II cytoskeletal	31	48	1.4 07	1.86 E-02	NA	NA
10	VINQTQKEN LR	11	1341. 737	g.17342	60S ribosomal protein L35	76	86	1.4 69	3.61 E-02	-0.740	1.23E-45
11	QVHPDTGIS	9	952.4 61	g.33075	Histone H2B 1.1	63	71	2.4 15	1.22 E-02	0.620	9.99E-01
12	AQGQRDLD RIAGQVAAA SKKH	21	2219. 188	g.67000	40S ribosomal protein S19	142	162	1.5 65	1.77 E-02	-1.946	2.01E-13
13	THTNKPGLIN AVR	13	1419. 795	g.68918	Collagen alpha-3(VI) chain	690	702	1.6 08	2.47 E-02	2.389	2.59E-01
14	KLNQLKPGL Q	10	1137. 687	g.11095	Interleukin enhancer-binding factor 3	409	418	2.6 26	2.47 E-02	0.634	2.32E-01
15	PEPAKSAPA PKKG	13	1276. 714	g.33075	Histone H2B 1.1	17	29	1.6 86	2.47 E-02	0.620	9.99E-01
16	AVQAEKSRS GSSRQSIQ	17	1817. 934	g.67758	Histone H1.0	35	51	3.3 27	2.47 E-02	-0.033	9.99E-01
17	TSGTLKQTK GVGASGSFR	18	1780. 943	g.67758	Histone H1.0	77	94	1.9 65	2.47 E-02	-0.033	9.99E-01
18	LDSIKGMAIS	17	1718.	g.17769	Brevinin-2Ef	73	89	1.8	2.47	-1.320	1.45E-08

	TGKSALQ		924					40	E-02		
19	TFSTASAVPANRNSFSSYSTVR	22	2349.135	g.65074	Keratin, type II cytoskeletal 75	50	71	1.816	3.32E-02	1.192	1.44E-05
20	KPLDLRPKKT	10	1194.745	g.17342	60S ribosomal protein L35	95	104	1.503	3.57E-02	-0.740	1.23E-45
21	ISGLIYEETRGVLK	14	1576.883	g.45818	Histone H4	47	60	1.615	3.61E-02	NA	NA
22	KHAVSEGTKAVTKYTSAK	18	1905.032	g.33075	Histone H2B 1.1	124	141	2.109	3.61E-02	0.620	9.99E-01
23	AENSSAAPAAKPKR	14	1396.742	g.67758	Histone H1.0	2	15	1.457	3.61E-02	-0.033	9.99E-01
24	QTKGVGASGSFR	12	1193.615	g.67758	Histone H1.0	83	94	1.707	3.92E-02	-0.033	9.99E-01
25	GAYKYMQELWR	11	1443.697	g.34359	60S ribosomal protein L15	2	12	2.135	3.92E-02	-0.717	1.81E-33
26	STITSREIQTAVRLL	15	1686.963	g.33075	Histone H2B 1.1	103	117	2.360	3.97E-02	0.620	9.99E-01
27	TKKSLESINSRLQ	13	1502.842	g.75113	60S ribosomal protein L30	4	16	1.497	3.97E-02	-0.862	2.06E-40

		**Peptide Quant**		**Downegulated**						**Transcript**	**Quant**
**Number**	**Sequence**	**Length**	**Mass**	**Leading Razor Protein**	**Protein Description**	**Start Position**	**End Position**	**Ratio**	**P value**	**Log2 Fold Change**	**Q Value Storey**

**1**	VSWFDQQN GRTYLK	14	1740.858	g.15884	Histone H1x	5	18	0.669	1.11E-03	-0.415	9.99E-01
**2**	LKGGTSLNI GKALDFVAK NQFVK	23	2447.390	g.68918	Collagen alpha-3(VI) chain	706	728	0.5664	2.33E-03	2.389	2.59E-01
3	NGNSYKALE SPSKNLLL	17	1846.979	g.10808	Nuclear factor 7, ovary	269	285	0.4115	2.33E-03	-1.992	1.61E-05
4	ALFRENLNK LM	11	1347.733	g.55252	Myosin-8	65	75	0.2885	2.69E-03	5.429	3.98E-143
5	SLGGGRSG GSYSVGVSS QSLYNLG	24	2288.103	g.64648	Unknown	145	168	0.3666	5.04E-03	NA	NA
6	RVLTVINQT QKENLR	15	1811.038	g.17342	60S ribosomal protein L35	72	86	0.586	6.15E-03	-0.740	1.23E-45
7	KLGNEQPVD TSTISLQ	16	1728.889	g.14919	Calponin-1	191	206	0.331	6.37E-03	1.094	7.68E-04
8	KVLKQVHPD TGISSKAMS	18	1925.040	g.33075	Histone H2B 1.1	59	76	0.682	1.07E-02	0.620	9.99E-01
9	ALNADDKAR	11	1241.	g.39795	Hemoglobin	2	12	0.3	1.37	-1.896	1.26E-11

	IR		684		subunit alpha-C			66	E-02		
10	AMDVVYALK	9	1008.531	g.45818	Histone H4	84	92	0.497	1.73 E-02	NA	NA
11	MEEQTQRPIK	10	1258.634	g.26291	Protein phosphatase inhibitor 2	1	10	0.738	1.73 E-02	0.012	9.99E-01
12	TGSERSLAKGSAAPGPVPQGLIRLY	25	2524.376	g.4260	Glutathione S-transferase omega-1	2	26	0.618	1.86 E-02	4.880	1.25E-03
13	GKKLQKVLLK	10	1153.791	g.80560	Low density lipoprotein receptor adapter protein 1-B	81	90	0.454	1.96 E-02	-1.897	9.99E-01
14	TETENAPAAAPPAEPAAKK	19	1862.937	g.34398	Histone H1A	14	32	0.695	1.96 E-02	-1.155	9.99E-01
15	LPGELAKHAVSEGTKAVTKYTSAK	24	2485.354	g.33075	Histone H2B 1.1	118	141	0.574	1.96 E-02	0.620	9.99E-01
16	ISGLKNVGKEVGMDVV	16	1643.892	g.81428	Esculentin-1B	83	98	0.703	2.47 E-02	0.073	1.61E-41
17	NLLISGLKNVGKEVGMDVVR	20	2140.204	g.81428	Esculentin-1B	80	99	0.338	2.47 E-02	0.073	1.61E-41
18	QLFNGTFVR	9	1080.572	g.67201	60S ribosomal protein L7	73	81	0.315	2.47 E-02	-0.982	6.12E-100

19	ISTGKSALQ NLLK	13	1371. 809	g.17769	Brevinin-2Ef	81	93	0.4 00	2.47 E-02	-1.320	1.45E-08
20	GMAISTGKS ALQNLLK	16	1630. 908	g.17769	Brevinin-2Ef	78	93	0.2 97	2.47 E-02	-1.320	1.45E-08
21	DNIQGITKPA IR	12	1324. 746	g.45818	Histone H4	25	36	0.6 32	3.32 E-02	NA	NA
22	EIRRYQKST ELLIR	14	1804. 032	g.77001	Histone H3.3	51	64	0.5 06	3.32 E-02	-0.948	5.14E-13
23	DNIQGITKPA IRRL	14	1593. 932	g.45818	Histone H4	25	38	0.4 49	3.61 E-02	NA	NA
24	SALTIEERAG FLN	13	1419. 736	g.8478	Non-muscle caldesmon	388	400	0.6 63	3.61 E-02	0.942	9.37E-01
25	FSNEPTSEF VLR	12	1424. 694	g.68918	Collagen alpha-3(VI) chain	678	689	0.6 92	3.77 E-02	2.389	2.59E-01
26	IFSLIKGAAK	10	1046. 649	g.35375	Unknown	5	14	0.6 30	3.92 E-02	-1.340	4.64E-81
27	IFSKLAGKKI K	11	1231. 802	g.81428	Esculentin-1B	69	79	0.3 71	3.97 E-02	0.073	1.61E-41

Table.S2 Differentially expressed metabolites in the four groups (Top 100)

Accession	IR_RIFSP-2_Mean	IR_Mean	RIFSP-2_Mean	CON_Mean	P.value	-log10 (P.value)	FDR	VIP	Class	mz
LPE(16:1)	331720.05	728060.6	1631409.08	442352.75	1.6442E-05	4.78	0.00795023	1.37705506	LPE	450.262616
PC(10:1e_8:0)	25992569.9	43856446.6	64350372.2	29810730.5	1.9524E-05	4.71	0.00795023	1.17010294	PC	522.355418
PG(16:0_16:0)	6693686.74	7868958.07	19795472.8	10456651.3	2.0147E-05	4.7	0.00795023	2.23519148	PG	721.502512
LPE(22:5)	628959.29	960841.8	1556212.37	817401	2.2244E-05	4.65	0.00795023	1.82249274	LPE	526.293916
LPC(18:1)	7217845.48	12356851.1	17028782.5	8002132.62	3.8491E-05	4.41	0.01028751	1.02320253	LPC	566.346346
PE(18:1_22:0)	44723199.6	49504429.6	45717393.9	39915332.7	4.3617E-05	4.36	0.01028751	1.15369378	PE	800.617481
LPC(19:1)	446667.57	531910.71	1169598.47	575526.5	6.1139E-05	4.21	0.01162781	1.77212997	LPC	536.371068
LPE(22:0)	710004.44	684676.08	907207.41	1028135.83	6.5051E-05	4.19	0.01162781	2.32217652	LPE	536.372166
Cer(m44:0+O)	1086250.73	1024603.73	2185912.81	1457108.53	8.5566E-05	4.07	0.01411839	1.77720143	Cer	724.682448

Cer(d44:0)	1099763.58	1060607.9	2197108.72	1509897.25	0.00010209	3.99	0.01564138	1.79040367	Cer	724.682448
WE(21:1)	1771575.03	7081718.41	5344527.31	3534707.64	0.00012097	3.92	0.01632949	1.21460516	WE	342.336655
Cer(m18:1_26:0+O)	5985143.37	5533510.61	10642462.4	7958696.75	0.00012829	3.89	0.01632949	1.97604473	Cer	678.67587
LPC(22:5)	490651.87	698319.01	1298821.21	612031.64	0.00012942	3.89	0.01632949	1.63617627	LPC	570.355418
PC(22:1)	69012.84	753679.06	1330589.97	386458.59	0.00013921	3.86	0.01658894	1.69095014	PC	592.397283
LdMePE(16:0)	232561.64	331855.26	651515.76	356571.05	0.00016511	3.78	0.01863958	2.01793174	LdMePE	480.309566
PMe(18:0_18:1)	326922.31	983081.12	1705707.97	768965	0.00018435	3.73	0.01977137	1.78847387	PMe	715.528332
Cer(d43:0)	660056.26	659172.03	933351.92	955738.4	0.00021867	3.66	0.02233554	2.35029697	Cer	710.666798
LPE(20:3)	153927.56	505317.26	769418.68	248935.36	0.00024255	3.62	0.02254504	1.2321969	LPE	502.293916
PE(18:1_22:1)	93832347.9	102632964	100077511	80839185.8	0.00026153	3.58	0.02254504	1.0162049	PE	798.601831
dMePE(20:1_18:1)	93832347.9	102632964	100077511	80839185.8	0.00026153	3.58	0.02254504	1.0162049	dMePE	798.601831
LPC(15:0)	279290.24	448135.3	787987.96	433131.41	0.00026935	3.57	0.02254504	1.90906395	LPC	482.324118
LPE(20:4)	1689575.93	3683563.74	5891639.31	2701574.54	0.00027439	3.56	0.02254504	1.72471996	LPE	500.278266

Cer(d18:1_24:1)	492686985	466755080	357052002	357514981	0.00028378	3.55	0.02254504	2.46086773	Cer	648.62892
LPE(18:1)	2650699.75	6619159.04	12250733.7	4442479.57	0.00031776	3.5	0.02369489	1.59691881	LPE	478.293916
PMe(16:0_16:1)	835042.86	1516341.01	2890819.57	1288025.44	0.00032514	3.49	0.02369489	1.45909946	PMe	659.465732
LPC(22:6)	410239.52	577932.02	1051931.09	461237.19	0.00034629	3.46	0.02396123	1.27817074	LPC	568.339768
PMe(16:0_18:1)	3407386.99	6819083.38	11479855.8	5587749.75	0.00036772	3.43	0.02464852	1.54764922	PMe	687.497032
LPC(14:0)	122537.6	261782.06	368824.11	172616.17	0.00042595	3.37	0.02728547	1.33982422	LPC	512.299396
PC(22:5_18:2)	3590085.75	5015302.71	7270499.25	6340995.38	0.0004325	3.36	0.02728547	2.5023327	PC	876.576011
SM(d18:1_18:0)	7540743.02	6497796.34	5084781.91	3434775.48	0.00046223	3.34	0.02832789	1.81368889	SM	731.606152
PS(42:3)	2501533.01	2653478.36	2313156.01	2160680.35	0.00051252	3.29	0.03053735	2.02273506	PS	868.607311
Cer(d18:2_23:1)	1781074.55	1233532.81	607891.23	729821.58	0.00057361	3.24	0.03185192	2.24928788	Cer	632.59762
dMePE(16:0_22:1)	45108889.8	49345255.9	45232560.6	39687199.5	0.00058815	3.23	0.03185192	1.28940226	dMePE	800.617481
MePC(43:0e)	11934380.4	10745939	9551412.01	8495946.13	0.00059809	3.22	0.03185192	2.54013951	MePC	910.759913
WE(23:1)	569152.54	1329086.68	1747557.84	832915.97	0.00060661	3.22	0.03185192	1.34425928	WE	370.367955

PEt(16:0_20:4)	1682478.15	2235510.18	1756589.83	2873833.55	0.00062978	3.2	0.03185192	1.04276163	PEt	723.497032
PC(37:1e)	464708922	513119971	519714900	647075461	0.00063599	3.2	0.03185192	2.08978868	PC	788.652768
PC(16:1_18:1)	928553912	1187418672	1122498413	1164514694	0.000668	3.18	0.03256505	1.29035111	PC	802.560361
PMe(14:0_18:2)	68211.04	352561.29	594540.62	270320.24	0.00072408	3.14	0.03451457	1.74939758	PMe	657.450082
PE(17:1_18:2)	5459565.35	7061518.61	8202103.02	7590038.65	0.00077518	3.11	0.03500972	2.02443235	PE	726.507931
PMe(16:0_16:0)	260423.2	968051.24	1735642.35	715708.2	0.00078343	3.11	0.03500972	1.75304974	PMe	661.481382
Cer(d40:2)	435750.79	523105.26	1029551.35	769931.73	0.00089215	3.05	0.03891354	2.25270147	Cer	664.588548
dMePE(18:0_18:1)	2456543.68	3803246.82	4111719.15	3457203.58	0.00090708	3.04	0.03891354	1.44995958	dMePE	772.586181
PMe(16:1_18:2)	298679.52	972786.41	1464534.76	840474.39	0.00098971	3	0.04162583	1.96486173	PMe	683.465732
PG(16:0_14:0)	354454.41	383309.46	1315459.6	730415.73	0.00106075	2.97	0.04375584	2.14166737	PG	693.471212
SM(d43:7)	3890093	3725660.36	4254385.69	5261058.89	0.00109267	2.96	0.04422235	1.60219029	SM	817.621802
PMe(16:0_14:0)	143963.85	341603.12	644216.66	284083.24	0.0011365	2.94	0.04514411	1.55753676	PMe	633.450082
SM(d18:1_24:1)	7305387636	7603389683	8146649532	7495079625	0.00122793	2.91	0.04703425	1.72260962	SM	813.684402

SM(d18:2_24:0)	7305387636	7603389683	8146649532	7495079625	0.00122793	2.91	0.04703425	1.72260962	SM	813.684402
Cer(d43:4)	15690146.7	13409548.7	9950713.92	9113163.3	0.00132946	2.88	0.04916702	2.46088105	Cer	658.61327
PMe(18:1_18:2)	2382727.35	6519153.73	8808737.16	5542751.18	0.00140749	2.85	0.05117065	1.89054265	PMe	711.497032
PG(34:2)	4791755.29	4758861.55	7432685.5	6031699.03	0.00156831	2.8	0.05514783	2.12914161	PG	769.499009
MGDG(16:0_20:3)	47118518.2	41924218.1	30162110.5	22431777.2	0.00166329	2.78	0.05656828	1.60284732	MGDG	779.567874
LPC(20:1)	545480.15	714671.52	1481790.19	648768.64	0.00170288	2.77	0.05656828	1.51116918	LPC	594.377646
OAHFA(36:1)	238299.96	694082.94	763997.06	491938.99	0.00172883	2.76	0.05656828	1.567749	OAHFA	563.504484
LPC(18:0)	72563633.7	56149942.1	65630457.3	136084828	0.00175275	2.76	0.05656828	1.24217067	LPC	524.371068
DG(42:5)	1857273.7	3146724.23	4104126.16	3089127.54	0.00176693	2.75	0.05656828	1.85064112	DG	716.61875
PE(18:0e_18:1)	17088497.9	19272192.4	20175908.6	20361867.6	0.00181804	2.74	0.05734849	1.65867657	PE	730.575616
PC(30:0e)	232427216	214769051	198099145	126940918	0.00201768	2.7	0.06177281	2.25753248	PC	692.558868
LPC(20:4)	679372.88	1192018.29	1364931.43	942000.37	0.00204469	2.69	0.06177281	1.57753145	LPC	588.330696
Cer(d18:1_22:0)	100182754	90157558	61913097.6	63690304.3	0.00208246	2.68	0.06179971	2.38653467	Cer	622.61327

LPE(20:1)	446212.32	640530.42	1140242.62	751961.73	0.00212054	2.67	0.06179971	1.92443748	LPE	506.325216
PC(26:0)	979924.55	1441026.01	1874329.35	1375588.06	0.00213202	2.67	0.06179971	1.81026605	PC	650.475533
Hex2Cer(d42:2)	1389352.24	1751624.53	2228098.76	1574423.33	0.00220178	2.66	0.06297093	1.24890335	Hex2Cer	1030.74115
SM(t38:5)	734033989	702696861	611191141	649948269	0.00224075	2.65	0.06324225	2.14356301	SM	767.569767
PE(18:1e)	3256922.92	5034662.67	9080117.98	5488883.86	0.00230557	2.64	0.06422651	2.23770185	PE	480.308468
PA(38:5e)	1584368.11	3149787.05	2508287.57	3325558.3	0.00254319	2.59	0.06915752	1.69642691	PA	707.502117
WE(25:2)	350821.78	753870.78	1511329.96	769854.6	0.00256206	2.59	0.06915752	1.80137467	WE	396.383605
WE(19:2)	2388632.82	5448890.82	3729230.87	4758357.08	0.00261154	2.58	0.06915752	1.25740824	WE	312.289705
PC(18:1_23:0)	785422.01	392193.01	371073.71	227979.25	0.00266107	2.57	0.06960959	2.28512542	PC	902.685561
LPE(24:0)	1132446.52	977182.2	1382701.1	1645254.93	0.00275365	2.56	0.07116371	1.80054733	LPE	564.403466
PC(20:0_18:2)	12169514.5	11579465.8	8269642.93	8316118.27	0.00294516	2.53	0.07520673	2.03536474	PC	858.622961
WE(21:2)	6880169.14	16024738.3	12752313.9	13839104.8	0.00316167	2.5	0.07978562	1.46257093	WE	340.321005
PE(18:1e_17:0)	1193465.42	1726165.11	1858976.41	1855079.71	0.00329532	2.48	0.08219131	2.29528958	PE	716.559966

PC(16:1e_22:5)	4879245.97	4640702.85	3779558.6	2940118.38	0.00342235	2.47	0.08437851	2.27534437	PC	836.581096
Cer(d18:1_24:0+O)	3597850.24	3669052.06	2514856.79	3067373.74	0.00348823	2.46	0.08502566	2.02618751	Cer	666.639485
Cer(d18:1_16:0)	83657085.7	82537858.2	63272127.1	65114887	0.00368936	2.43	0.08873512	2.54570437	Cer	538.51937
PEt(17:1_18:1)	2679933.73	4281743.37	7470086.04	3628927.21	0.00376452	2.42	0.08873512	1.33140778	PEt	713.512682
PMe(18:1_18:1)	2679933.73	4281743.37	7470086.04	3628927.21	0.00376452	2.42	0.08873512	1.33140778	PMe	713.512682
PG(16:0_18:2)	21436213.6	28029472.8	41008373.5	36259257.3	0.00381381	2.42	0.08891984	2.11997111	PG	745.502512
WE(23:2)	16395037.5	41399799	30204479.9	35370709.5	0.00398419	2.4	0.09135901	1.43396349	WE	368.352305
Cer(d18:0_26:0)	3986334.56	4305875.96	6866526.89	4841311.26	0.00426898	2.37	0.09592082	1.50317938	Cer	680.69152
PS(49:2)	1819917.88	2401093	3060356.77	2353411.64	0.00429296	2.37	0.09592082	1.03390493	PS	968.732511
MePC(33:1e)	262136706	252736886	218836371	242766353	0.00441626	2.35	0.09765852	2.07010931	MePC	768.587763
MGDG(15:0_14:0)	3003613.09	5600299.42	4671941.89	5763472.55	0.00459724	2.34	0.0990654	1.73145728	MGDG	733.510754
PEt(16:1e_20:4)	1581617.97	3117484.19	2375058.83	3336650.66	0.00465673	2.33	0.0990654	1.55930711	PEt	707.502117
SM(d18:1_21:1)	15294558.6	18869679.5	23654033.7	17481374.9	0.00466462	2.33	0.0990654	1.61944159	SM	771.637452

AcCa(22:4)	1913643.06	3917930.11	2712139.76	6250739.65	0.0049137	2.31	0.10264129	1.5217231	AcCa	476.373435
DG(18:0_18:1)	379623847	505794019	461347581	454868807	0.00502191	2.3	0.10264129	1.27694316	DG	640.58745
PMe(16:0_18:2)	940161.21	1819733.69	3704846.86	1635782.31	0.0050244	2.3	0.10264129	1.47362211	PMe	685.481382
SPH(t17:0)	40687164.4	27249884.4	6829347.66	16537616	0.00512115	2.29	0.10363078	2.03701964	SPH	304.28462
LPE(22:6)	544046.08	643532.64	1398459.4	574885.75	0.00533184	2.27	0.10688602	1.31575327	LPE	524.278266
PC(28:1)	2629080.89	3382698.73	5722819.92	4597985.37	0.00550184	2.26	0.10827006	2.15087251	PC	676.491183
PE(16:0_14:0)	522077.14	902811.96	1474020.52	611785.1	0.00588535	2.23	0.11261718	1.12670018	PE	662.476631
CL(74:3)	2041779.78	2597689.25	3089265.34	2647471.31	0.00590708	2.23	0.11261718	2.01512537	CL	1486.0745
LPC(16:0)	7732237.57	9179407.63	15542197.9	12355635.5	0.00593275	2.23	0.11261718	2.07156576	LPC	540.330696
PE(16:1e_18:3)	3751624.3	5576973.42	6120333.4	5791705.5	0.00599926	2.22	0.11288072	1.94166456	PE	696.497366
PC(48:7)	1537822.37	2033664.32	2902239.53	2679011.86	0.00613789	2.21	0.11361848	2.51742322	PC	944.710283
LPE(22:4)	442503.18	942089.66	1145275.93	902440.1	0.0061444	2.21	0.11361848	1.91687379	LPE	528.309566
MePC(38:0)	13172771.5	13311142.1	17031114.3	14268056.3	0.00631706	2.2	0.11450771	1.66380349	MePC	854.660928

Table.S3 Differentially expressed metabolites in irradiated HaCaT cells pretreated with RIFSP-2 or not (Top 100)

Accession	IR_RIFSP-2_Mean	IR_Mean	FC	log2FC	P.value	-Log10 (P.value)	FDR	VIP	Class	mz	Regulation
PC(16:1_18:1)	928553912	1187418672	0.78	-0.35	7.2142E-05	4.14	0.09522002	2.07969869	PC	802.560361	Down
LPE(22:5)	628959.29	960841.8	0.65	-0.61	8.8783E-05	4.05	0.09522002	2.06648517	LPE	526.293916	Down
PE(18:0e_18:1)	17088497.9	19272192.4	0.89	-0.17	0.00059948	3.22	0.22354226	1.94317766	PE	730.575616	Down
MGMG(27:0)	1746208.98	2052054.57	0.85	-0.23	0.00065201	3.19	0.22354226	1.95405124	MGMG	705.515839	Down
MePC(29:1e)	24314619.3	21409219.6	1.14	0.18	0.00071972	3.14	0.22354226	1.9789821	MePC	712.525163	Up
PC(19:1)	2518924.64	3258359.51	0.77	-0.37	0.00106228	2.97	0.22354226	1.93764794	PC	550.350333	Down
MGDG(34:4e)	4377393.8	6939668.36	0.63	-0.66	0.00107283	2.97	0.22354226	1.93013559	MGDG	735.541659	Down
PE(17:1_18:1)	25800475.1	35669291.8	0.72	-0.47	0.00113967	2.94	0.22354226	1.97816414	PE	728.523581	Down
WE(21:1)	1771575.03	7081718.41	0.25	-2	0.00119513	2.92	0.22354226	1.94782011	WE	342.336655	Down

PEt(36:2e)	536480.39	1811575.83	0.3	-1.76	0.00121308	2.92	0.22354226	1.96086586	PEt	713.549067	Down
PC(16:0_24:1)	9401127.87	11118255.1	0.85	-0.24	0.00139706	2.85	0.22354226	1.88411469	PC	888.669911	Down
OAHFA(36:1)	238299.96	694082.94	0.34	-1.54	0.00163912	2.79	0.22354226	1.8968999	OAHFA	563.504484	Down
PE(18:1e_17:0)	1193465.42	1726165.11	0.69	-0.53	0.00210591	2.68	0.22354226	1.85020223	PE	716.559966	Down
PC(16:0_17:0)	188646009	218228182	0.86	-0.21	0.0024513	2.61	0.22354226	1.86691867	PC	806.591661	Down
PC(32:4e)	24575446.6	21276491.9	1.16	0.21	0.00245717	2.61	0.22354226	1.8818777	PC	712.527568	Up
TG(18:1_20:4_24:1)	16940117	12745853	1.33	0.41	0.00264413	2.58	0.22354226	1.88458185	TG	991.868816	Up
PC(14:1e_20:2)	16666094.1	24964778.9	0.67	-0.58	0.00275461	2.56	0.22354226	1.87557232	PC	742.574518	Down
LPC(17:1)	612058.68	925255.14	0.66	-0.6	0.00285973	2.54	0.22354226	1.8467833	LPC	508.339768	Down
CerG2GNAc1(d40:1)	3687520.48	4741381.66	0.78	-0.36	0.00288987	2.54	0.22354226	1.85694222	CerG2GNAc1	1149.79829	Down
PE(20:3_18:2)	3947783.45	5700275.72	0.69	-0.53	0.00290951	2.54	0.22354226	1.87442383	PE	764.523581	Down
PC(36:4)	1041383103	917659326	1.13	0.18	0.0029629	2.53	0.22354226	1.86022387	PC	782.569433	Up
PS(38:4e)	8107634.1	9350804.7	0.87	-0.21	0.00318118	2.5	0.22354226	1.8535532	PS	796.549796	Down

Cer(d30:3)	263670.19	713786.16	0.37	-1.44	0.00328775	2.48	0.22354226	1.85920547	Cer	478.42547	Down
PC(29:4e)	514527.18	397138.15	1.3	0.37	0.00331129	2.48	0.22354226	1.86478649	PC	670.480618	Up
PC(18:1_20:2)	218490127	272261694	0.8	-0.32	0.00331571	2.48	0.22354226	1.84168659	PC	856.607311	Down
PC(22:1)	69012.84	753679.06	0.09	-3.45	0.00334991	2.47	0.22354226	1.83505352	PC	592.397283	Down
PC(40:0e)	6089747.02	7263714.78	0.84	-0.25	0.00336696	2.47	0.22354226	1.82369353	PC	832.715368	Down
PC(28:1_18:1)	1221558.66	1709441.18	0.71	-0.48	0.00373447	2.43	0.22354226	1.7921196	PC	970.748161	Down
MGMG(28:0)	112337896	132690269	0.85	-0.24	0.00382873	2.42	0.22354226	1.82197872	MGMG	719.531489	Down
CL(76:0)	278652.37	497485.15	0.56	-0.84	0.00423155	2.37	0.22354226	1.7946859	CL	1520.15275	Down
WE(19:2)	2388632.82	5448890.82	0.44	-1.19	0.00444542	2.35	0.22354226	1.82461321	WE	312.289705	Down
MGDG(34:2e)	1332277.02	1936459.07	0.69	-0.54	0.00444801	2.35	0.22354226	1.78525207	MGDG	785.578439	Down
PS(32:0e)	6389939.38	8717847.27	0.73	-0.45	0.00453637	2.34	0.22354226	1.80651133	PS	720.518496	Down
PC(16:1e_20:1)	1755866.78	2914364.85	0.6	-0.73	0.00461719	2.34	0.22354226	1.79785691	PC	830.628046	Down
PC(10:1e_8:0)	25992569.9	43856446.6	0.59	-0.75	0.0046942	2.33	0.22354226	1.80162401	PC	522.355418	Down

SM(t34:1)	38127890.1	42285545	0.9	-0.15	0.00474786	2.32	0.22354226	1.82649718	SM	719.569767	Down
SM(d32:1)	112166146	132320866	0.85	-0.24	0.00477442	2.32	0.22354226	1.79731175	SM	719.53448	Down
PE(16:0_17:0)	852553.42	1237892.17	0.69	-0.54	0.00477928	2.32	0.22354226	1.79103693	PE	704.523581	Down
dMePE(15:0_16:0)	852553.42	1237892.17	0.69	-0.54	0.00477928	2.32	0.22354226	1.79103693	dMePE	704.523581	Down
Hex2Cer(d18:1_16:0+O)	5423227.69	7114583.41	0.76	-0.39	0.00477957	2.32	0.22354226	1.78321054	Hex2Cer	878.619935	Down
PC(42:1e)	12299738.1	15747774.5	0.78	-0.36	0.00485519	2.31	0.22354226	1.8092765	PC	858.731018	Down
LPC(22:5)	490651.87	698319.01	0.7	-0.51	0.00522685	2.28	0.22354226	1.81023318	LPC	570.355418	Down
DG(18:0_18:1)	379623847	505794019	0.75	-0.41	0.00534516	2.27	0.22354226	1.78587051	DG	640.58745	Down
MGDG(38:3e)	3614718.1	6158527.62	0.59	-0.77	0.00556786	2.25	0.22354226	1.7944933	MGDG	853.641039	Down
TG(18:1_20:2_20:2)	266483316	133484412	2	1	0.00563312	2.25	0.22354226	1.80114256	TG	954.848415	Up
LPE(20:3)	153927.56	505317.26	0.3	-1.71	0.0058131	2.24	0.22354226	1.77660821	LPE	502.293916	Down
TG(18:1_18:2_24:2)	11775190.4	9070612.34	1.3	0.38	0.00584492	2.23	0.22354226	1.79796047	TG	965.853166	Up
TG(18:1_18:1_20:4)	102820607	82764065.4	1.24	0.31	0.00587733	2.23	0.22354226	1.7612805	TG	907.774916	Up

TG(12:1e_6:0_22:6)	82620092.2	100550144	0.82	-0.28	0.00597487	2.22	0.22354226	1.79568696	TG	667.529601	Down
PE(37:1e)	11181330.2	23854408	0.47	-1.09	0.006073	2.22	0.22354226	1.77000677	PE	768.587763	Down
CL(74:3)	2041779.78	2597689.25	0.79	-0.35	0.00610001	2.21	0.22354226	1.753032	CL	1486.0745	Down
MGDG(15:0_14:0)	3003613.09	5600299.42	0.54	-0.9	0.00611604	2.21	0.22354226	1.82368702	MGDG	733.510754	Down
LPE(16:1)	331720.05	728060.6	0.46	-1.13	0.00625339	2.2	0.22354226	1.74839635	LPE	450.262616	Down
MePC(34:5)	12498523.5	10610286.9	1.18	0.24	0.00626293	2.2	0.22354226	1.79345336	MePC	788.520078	Up
PE(18:1_20:4)	12498523.5	10610286.9	1.18	0.24	0.00626293	2.2	0.22354226	1.79345336	PE	788.520078	Up
LPC(18:1)	7217845.48	12356851.1	0.58	-0.78	0.00633436	2.2	0.22354226	1.76191684	LPC	566.346346	Down
DG(20:0_18:1)	20168765.9	28349997	0.71	-0.49	0.00638996	2.19	0.22354226	1.76199371	DG	668.61875	Down
PC(39:4e)	21206356.9	29183638.4	0.73	-0.46	0.00648203	2.19	0.22354226	1.74637172	PC	810.637118	Down
PC(26:0)	979924.55	1441026.01	0.68	-0.56	0.0064842	2.19	0.22354226	1.75792439	PC	650.475533	Down
SM(t34:0)	15828732	19377332.8	0.82	-0.29	0.00652987	2.19	0.22354226	1.7771601	SM	721.585417	Down
PMe(35:2)	9597143.39	19689411.5	0.49	-1.04	0.00659917	2.18	0.22354226	1.78251947	PMe	699.497032	Down

TG(18:1_20:4_24:0)	10319061.9	7878301.35	1.31	0.39	0.00665138	2.18	0.22354226	1.77045613	TG	993.884466	Up
PG(18:1_20:2)	2753000.84	3537264.67	0.78	-0.36	0.00684561	2.16	0.22354226	1.7558275	PG	799.549462	Down
PC(18:0_16:0)	184750972	216390731	0.85	-0.23	0.00688337	2.16	0.22354226	1.74663475	PC	806.591661	Down
LPE(20:4)	1689575.93	3683563.74	0.46	-1.12	0.00689586	2.16	0.22354226	1.74641743	LPE	500.278266	Down
LPE(18:1)	2650699.75	6619159.04	0.4	-1.32	0.00692323	2.16	0.22354226	1.74302476	LPE	478.293916	Down
PC(14:1e_22:3)	11009681.3	23685047	0.46	-1.11	0.00705729	2.15	0.22354226	1.75336221	PC	768.590168	Down
PC(36:3)	1.8749E+10	1.8219E+10	1.03	0.04	0.00709201	2.15	0.22354226	1.77931594	PC	784.585083	Up
PEt(38:5)	8616896.58	14723486.4	0.59	-0.77	0.00748045	2.13	0.22354226	1.76943688	PEt	749.512682	Down
dMePE(18:0_18:1)	2456543.68	3803246.82	0.65	-0.63	0.00748322	2.13	0.22354226	1.74432659	dMePE	772.586181	Down
MePC(34:3)	505591116	461401261	1.1	0.13	0.00748946	2.13	0.22354226	1.72597226	MePC	787.595982	Up
LPC(16:1)	1311938.9	1897387.8	0.69	-0.53	0.00750352	2.12	0.22354226	1.73117904	LPC	538.315046	Down
PS(18:1_22:0)	2373381.61	3851175.62	0.62	-0.7	0.00768734	2.11	0.2258814	1.73701152	PS	844.607311	Down
MGDG(36:2e)	6826935.15	10370699	0.66	-0.6	0.00790691	2.1	0.22831472	1.74322967	MGDG	827.625389	Down

TG(26:1_16:0_18:1)	10354925.6	7963250.41	1.3	0.38	0.00803978	2.09	0.22831472	1.74572819	TG	993.882061	Up
MGDG(14:1e_16:0)	65194630.3	77172048.2	0.84	-0.24	0.00813869	2.09	0.22831472	1.73210052	MGDG	745.547139	Down
TG(4:0_16:0_18:1)	886954.21	2176813.07	0.41	-1.3	0.00819591	2.09	0.22831472	1.73754708	TG	682.598015	Down
CL(81:4)	1360467.09	2243155.18	0.61	-0.72	0.00840875	2.08	0.23124073	1.73845043	CL	1582.1684	Down
PG(36:1e)	23818597.8	28562313	0.83	-0.26	0.00853715	2.07	0.23179995	1.71866698	PG	761.570197	Down
MePC(33:6)	212609291	174729941	1.22	0.28	0.00875524	2.06	0.2347499	1.73137476	MePC	767.533382	Up
DG(18:1_24:0)	15863647.8	22617426.2	0.7	-0.51	0.00914114	2.04	0.23658796	1.71614221	DG	724.68135	Down
PC(16:1e_16:0)	137087554	162030132	0.85	-0.24	0.00927002	2.03	0.23658796	1.7161873	PC	762.565446	Down
PC(16:0_24:0)	1727283.62	2062365.57	0.84	-0.26	0.00928481	2.03	0.23658796	1.69738026	PC	890.685561	Down
DG(18:1_22:1)	31152629	48019120.3	0.65	-0.62	0.00944282	2.02	0.23658796	1.72170333	DG	694.6344	Down
PG(18:1_20:4)	12659985.7	16128200.9	0.78	-0.35	0.00989425	2	0.23658796	1.71319798	PG	795.518162	Down
DG(17:0_18:1)	14969906.2	19339920.1	0.77	-0.37	0.00997784	2	0.23658796	1.71605769	DG	626.5718	Down
DGDG(36:1e)	2125439.71	2509652.54	0.85	-0.24	0.00998444	2	0.23658796	1.67891968	DGDG	931.672734	Down

WE(23:2)	16395037.5	41399799	0.4	-1.34	0.010559<br>71	1.98	0.236587<br>96	1.720973<br>12	WE	368.3523<br>05	Down
PEt(17:1_18:2)	1161230.97	2137256.<br>54	0.54	-0.88	0.010757<br>51	1.97	0.236587<br>96	1.712704<br>58	PEt	711.4970<br>32	Down
WE(21:2)	6880169.14	16024738<br>.3	0.43	-1.22	0.010777<br>77	1.97	0.236587<br>96	1.716833<br>59	WE	340.3210<br>05	Down
PMe(18:0_18:1)	326922.31	983081.1<br>2	0.33	-1.59	0.010977<br>01	1.96	0.236587<br>96	1.715930<br>14	PMe	715.5283<br>32	Down
PEt(17:1_16:1)	1838796.81	2760224.<br>08	0.67	-0.59	0.011149<br>95	1.95	0.236587<br>96	1.733039<br>05	PEt	685.4813<br>82	Down
PE(17:1_18:2)	5459565.35	7061518.<br>61	0.77	-0.37	0.011369<br>4	1.94	0.236587<br>96	1.694776<br>37	PE	726.5079<br>31	Down
PE(16:1e_18:3)	3751624.3	5576973.<br>42	0.67	-0.57	0.011404<br>9	1.94	0.236587<br>96	1.677212<br>35	PE	696.4973<br>66	Down
PEt(18:1_18:1)	45188343.5	66279157	0.68	-0.55	0.011454<br>79	1.94	0.236587<br>96	1.739724<br>71	PEt	727.5283<br>32	Down
MGDG(30:0e)	383698.16	1063110.<br>97	0.36	-1.47	0.011486<br>46	1.94	0.236587<br>96	1.726307<br>35	MGDG	687.5416<br>59	Down
DG(16:0_24:1)	6999465.9	10047315<br>.5	0.7	-0.52	0.011536<br>53	1.94	0.236587<br>96	1.664552<br>69	DG	696.6500<br>5	Down
PC(14:1e_14:0)	6399423.66	8665945.<br>23	0.74	-0.44	0.011598<br>14	1.94	0.236587<br>96	1.824244<br>85	PC	720.5184<br>96	Down
Hex1Cer(t32:0)	2296210.66	2864747.<br>58	0.8	-0.32	0.011645<br>09	1.93	0.236587<br>96	1.670200<br>99	Hex1Cer	688.5369<br>08	Down
DG(18:0_20:4)	318787126	42513484<br>8	0.75	-0.42	0.011739<br>86	1.93	0.236587<br>96	1.702073<br>2	DG	662.5718	Down

Figure. S1

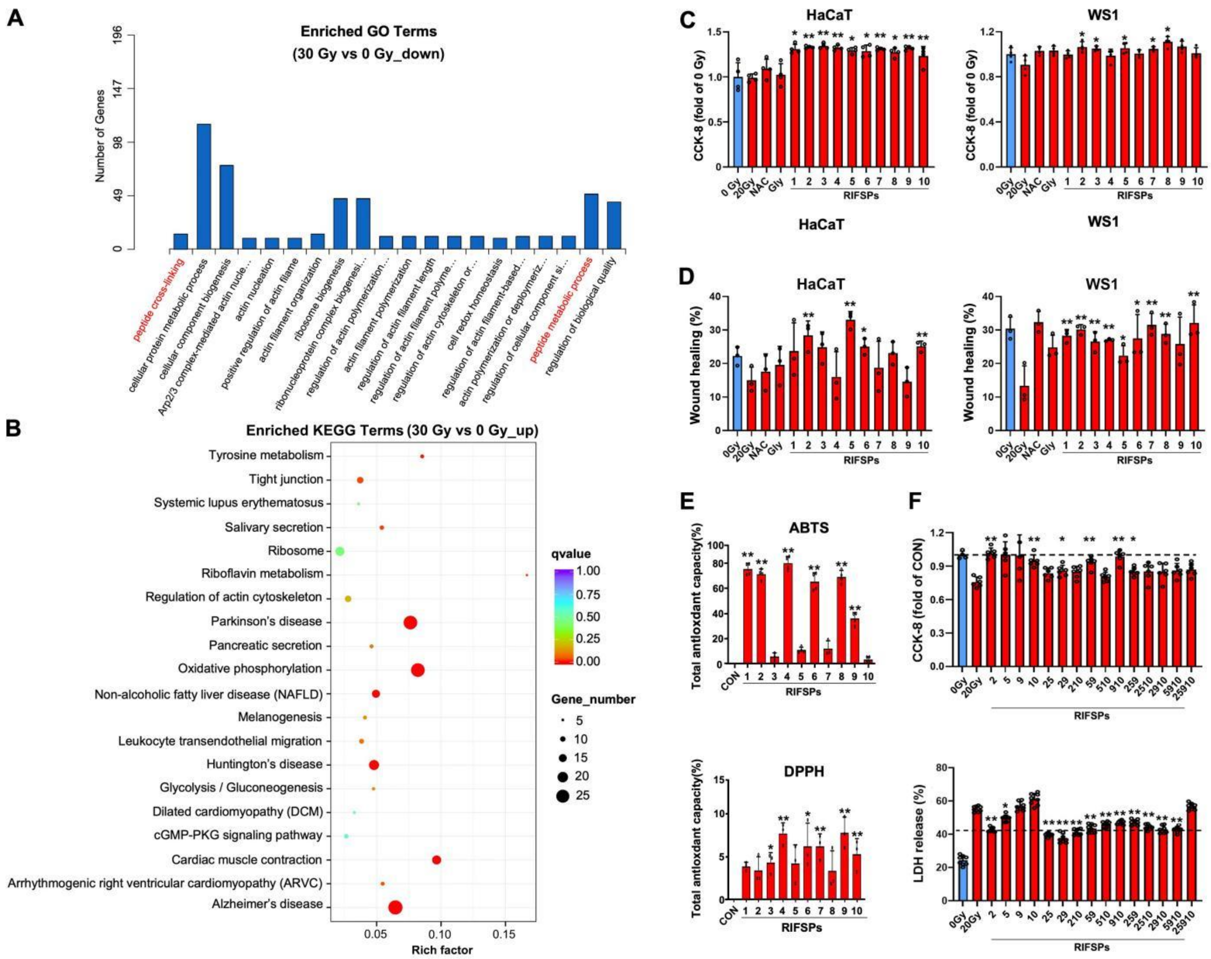

A
Enriched GO Terms
(30 Gy vs 0 Gy_down)
Number of Genes
peptide cross-linking
cellular protein metabolic process
cellular component biogenesis
actin nucleation
positive regulation of actin filame
actin filament organization
ribosome biogenesis
actin filament polymerization
regulation of actin filament length
cell redox homeostasis
peptide metabolic process
regulation of biological quality
B
Enriched KEGG Terms (30 Gy vs 0 Gy_up)
Tyrosine metabolism
Tight junction
Systemic lupus erythematosus
Salivary secretion
Ribosome
Riboflavin metabolism
Regulation of actin cytoskeleton
Parkinson's disease
Pancreatic secretion
Oxidative phosphorylation
Non-alcoholic fatty liver disease (NAFLD)
Melanogenesis
Leukocyte transendothelial migration
Huntington's disease
Glycolysis / Gluconeogenesis
Dilated cardiomyopathy (DCM)
cGMP-PKG signaling pathway
Cardiac muscle contraction
Arrhythmogenic right ventricular cardiomyopathy (ARVC)
Alzheimer's disease
Rich factor
qvalue
Gene_number
C
HaCaT
WS1
CCK-8 (fold of 0 Gy)
RIFSPs
D
HaCaT
WS1
Wound healing (%)
RIFSPs
E
ABTS
DPPH
Total antioxdant capacity(%)
RIFSPs
F
CCK-8 (fold of CON)
LDH release (%)
RIFSPs

Fig. S1 Investigations on the effects of RIFSPs in cell proliferation, migration, ROS elimination and pyroptosis in irradiated skin cells.

Gene Ontology (GO) analysis (A) and Kyoto Encyclopedia of Genes and Genomes (B) analysis of the differentially expressed genes in frog skin tissues induced by irradiation. Effect of the 10 most significantly upregulated RIFSPs on the proliferation, migration and total antioxidant ability of irradiated human skin cells, as detected through (C) CCK-8, (D) wound healing (E) DPPH and ABTS assays (n = 3, * $P < 0.05$; ** $P < 0.01$). (F) Investigation on the potential synergy effects among RIFSP-2/5/9/10 in irradiated HaCaT cells through CCK-8 and LDH assays (n = 6,

* $P < 0.05$; ** $P < 0.01$).

Figure. S2

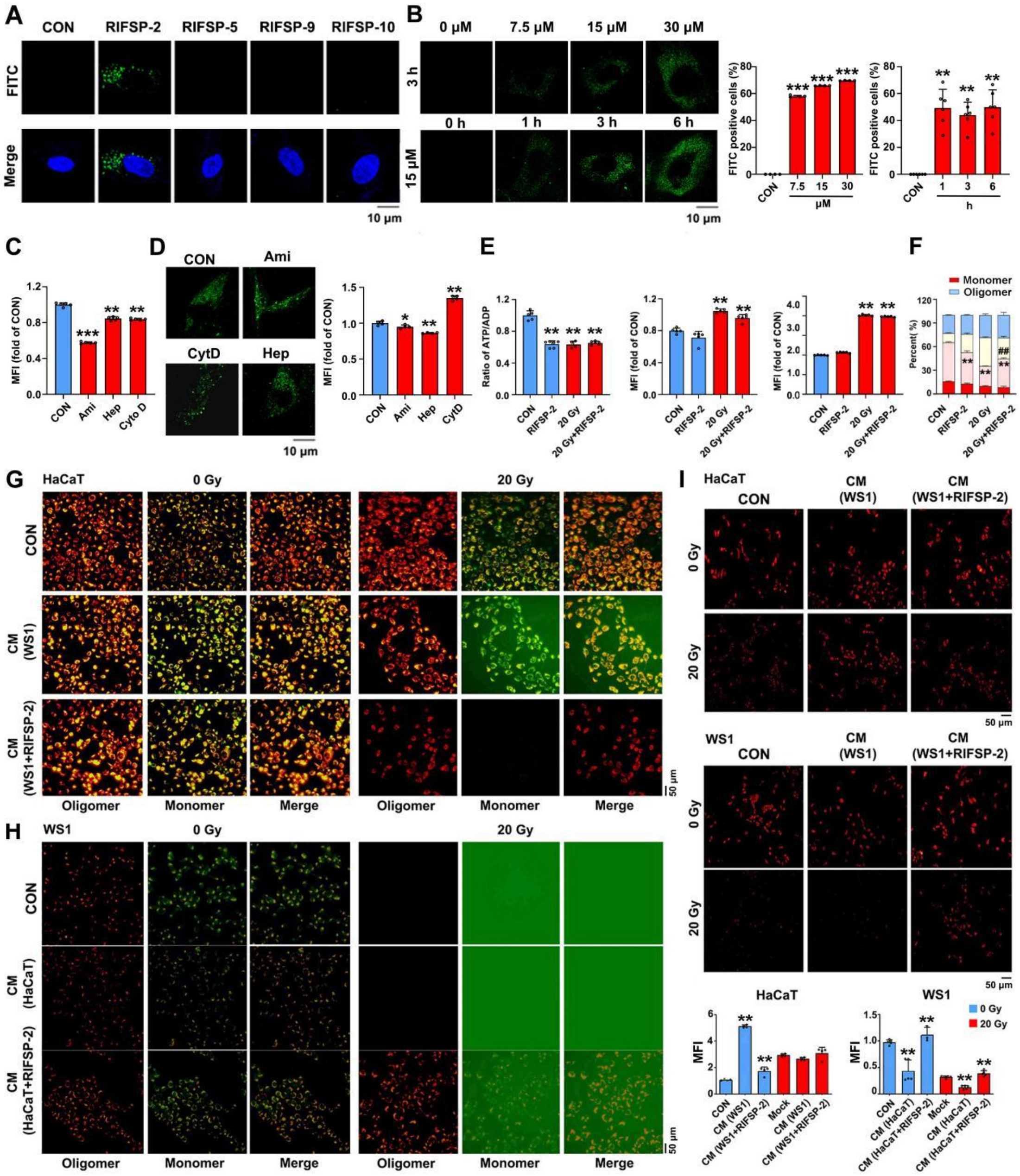
A
CON
RIFSP-2
RIFSP-5
RIFSP-9
RIFSP-10
FITC
Merge
10 μm
B
0 μM
7.5 μM
15 μM
30 μM
3 h
0 h
1 h
3 h
6 h
15 μM
10 μm
FITC positive cells (%)
CON
7.5
15
30
μM
h
C
MFI (fold of CON)
CON
Ami
Hep
Cyto D
D
CON
Ami
CytD
Hep
10 μm
E
Ratio of ATP/ADP
RIFSP-2
20 Gy
20 Gy+RIFSP-2
F
Monomer
Oligomer
Percent(%)
G
HaCaT
0 Gy
20 Gy
CM (WS1)
CM (WS1+RIFSP-2)
Oligomer
Monomer
Merge
50 μm
H
WS1
CM (HaCaT)
CM (HaCaT+RIFSP-2)
I
50 μm
MFI
0 Gy
20 Gy
Mock

Fig. S2 RIFSP-2 maintains homeostasis of mitochondria in irradiated skin cells.

(A) Administration of FITC-labeled RIFSPs to WS1 cells and identification of the membrane impermeability of RIFSP-2 through confocal microscopy. (B)Time- and dose-dependent uptake of FITC-RIFSP-2 by WS1 cells detected through confocal microscopy and flow cytometry analysis. (C) Influence of heparin, amiloride and cytochalasin D on the uptake of FITC-RIFSP-2 by HaCaT cells through flow cytometry analysis. (E) Influence of RIFSP-2 on mitochondrial ROS accumulation, energy production and cellular ROS accumulation through MitoSox probe staining, ADP probe staining and DCFH-DA staining, respectively (n = 4, ** $P < 0.01$). (F) Influence of RIFSP-2 on the mitochondrial membrane potential in irradiated HaCaT cells through JC-1staining. The different colors from blue to red represent the aggregate state of JC-1, from total monomer, partial oligomer, partial monomer to total oligomer. Conditioned medium from WS1 cells pretreated with RIFSP-2 increased MMP of irradiated HaCaT cells and vice versa, detected by JC-1(G and H) and Mito-Tracker Red CMXRos (I) staining (n = 4, ** $P < 0.01$).

Figure. S3

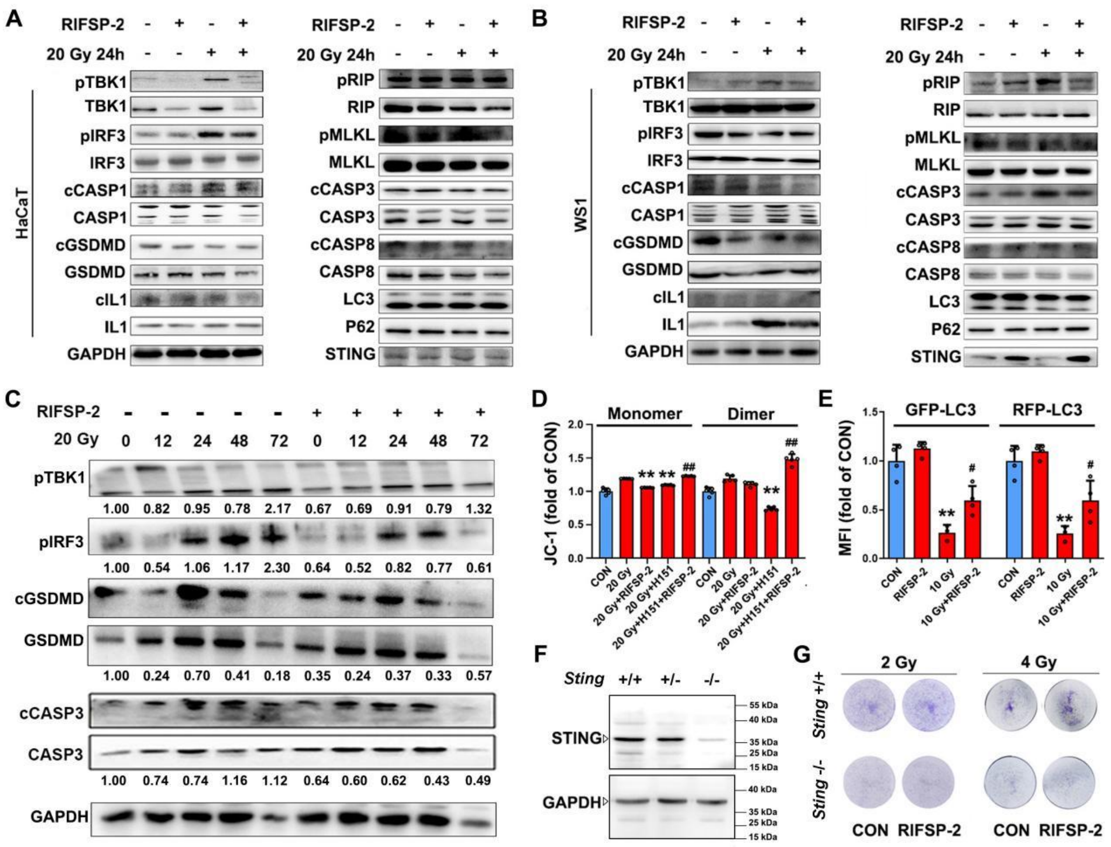

Fig. S3 Influence of RIFSP-2 on cell death and autophagy in irradiated skin cells.

Alterations in the expression of protein markers for pyroptosis, apoptosis, necroptosis and

ferroptosis in HaCaT (A), WS1 (B) and primary mice skin cells (C) after 20 Gy irradiation with RIFSP-2 pretreatment detected by Western blotting analysis. (D) Influence of STING antagonist, H151 in mitochondrial membrane potential of irradiated HaCaT cells pretreated with RIFSP-2 (n = 4, ** $P < 0.01$). (E) Flow cytometry analysis of autophagy by mRFP-GFP-LC3 in irradiated WS1 cells pretreated with 15 μM RIFSP-2 (n = 4, * $P < 0.05$; ** $P < 0.01$). (F) Identification of STING protein profiles of wild-type *Sting* ($Sting^{+/+}$), heterozygous ($Sting^{+/-}$) and *Sting* deficient ($Sting^{-/-}$) mice through Western blotting analysis. (G) Colony formation analysis of primary skin cells from wild-type *Sting* ($Sting^{+/+}$) and *Sting* deficient ($Sting^{-/-}$) mice exposed to single 2 Gy or 4 Gy irradiation, pretreated with RIFSP-2 or not (n = 3).

Figure. S4

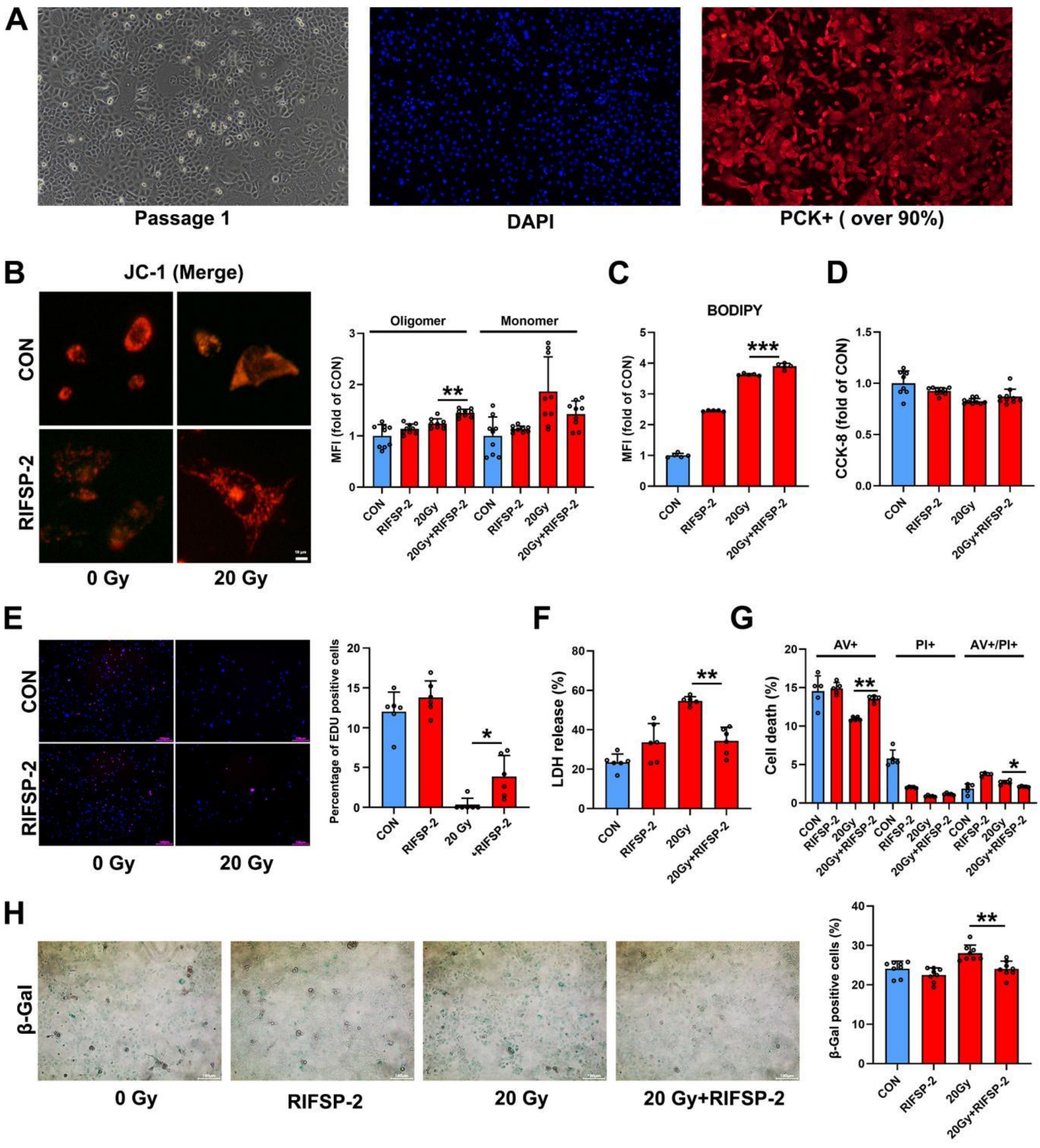
A
Passage 1
DAPI
PCK+ (over 90%)
B
JC-1 (Merge)
CON
RIFSP-2
0 Gy
20 Gy
Oligomer
Monomer
MFI (fold of CON)
**
CON
RIFSP-2
20Gy
20Gy+RIFSP-2
C
BODIPY
MFI (fold of CON)
***
D
CCK-8 (fold of CON)
E
CON
RIFSP-2
0 Gy
20 Gy
Percentage of EDU positive cells
*
20 Gy
+RIFSP-2
F
LDH release (%)
**
G
AV+
PI+
AV+/PI+
Cell death (%)
**
*
H
β-Gal
0 Gy
RIFSP-2
20 Gy
20 Gy+RIFSP-2
β-Gal positive cells (%)
**

Fig. S4 Protective effects of RIFSP-2 in primary human epidermal keratinocytes

(A) Observation of the typical morphological character of first passage human primary epidermal keratinocyts (HEKs) through phase contrast microscope and immunofluorescence assay for the ratio of PCK positive cells in first passage HEKs, with specific antibody against Pan-Cytokeratin (Proteintech). The effect of RIFSP-2 on mitochondrial membrane potential (B), neutral lipid droplet formation (C), cell viability (D) and cell proliferation (E) of third or fouth passages of HEKs, as detected by JC-1 staining, BODIPY staining, CCK-8 assays and Edu staining, respectively. (F and G) LDH release and AV/PI staining assays were adopted to investigate the effects of RIFSP-2 in radiation-induced death of fourth passages of HEKs (*$P < 0.05$ and **$P < 0.01$, compared with the 20 Gy group). (H) Influence of RIFSP-2 in radiation-induced senescence in second passage of HEKs, as detected by SA-β-Gal staining analysis (n = 6, ** $P < 0.01$).

Figure. S5

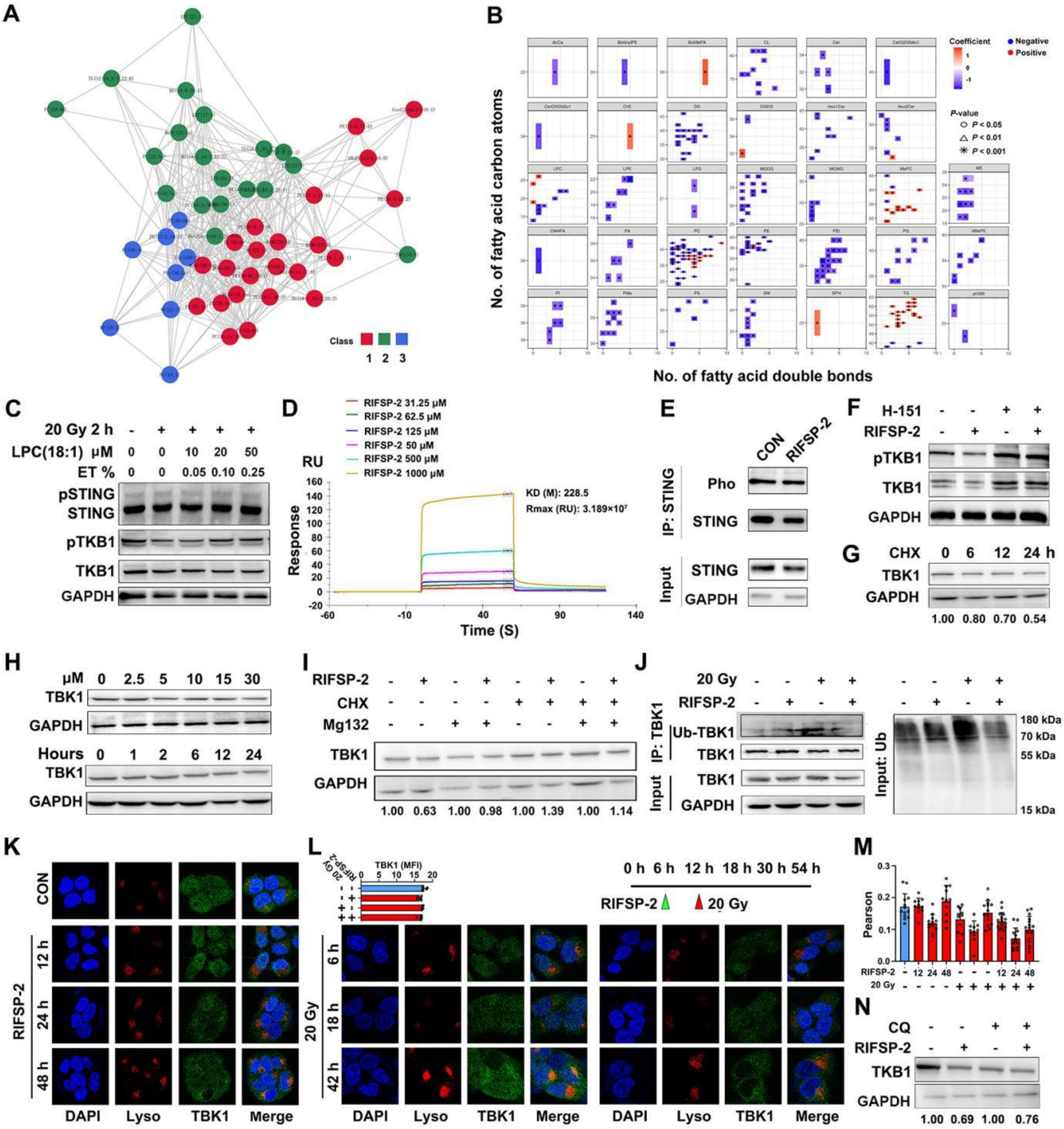
A
Class
1 2 3
B
Coefficient
Negative
Positive
P-value
P < 0.05
P < 0.01
P < 0.001
No. of fatty acid carbon atoms
No. of fatty acid double bonds
C
20 Gy 2 h
LPC(18:1) μM
ET %
pSTING
STING
pTKB1
TKB1
GAPDH
D
RIFSP-2 31.25 μM
RIFSP-2 62.5 μM
RIFSP-2 125 μM
RIFSP-2 50 μM
RIFSP-2 500 μM
RIFSP-2 1000 μM
RU
KD (M): 228.5
Rmax (RU): 3.189×10⁷
Response
Time (S)
E
CON
RIFSP-2
Pho
STING
IP: STING
Input
GAPDH
F
H-151
RIFSP-2
pTKB1
TKB1
GAPDH
G
CHX 0 6 12 24 h
TBK1
GAPDH
1.00 0.80 0.70 0.54
H
μM 0 2.5 5 10 15 30
TBK1
GAPDH
Hours 0 1 2 6 12 24
TBK1
GAPDH
I
RIFSP-2
CHX
Mg132
TBK1
GAPDH
1.00 0.63 1.00 0.98 1.00 1.39 1.00 1.14
J
20 Gy
RIFSP-2
IP: TBK1
Ub-TBK1
TBK1
Input
TBK1
GAPDH
Input: Ub
180 kDa
70 kDa
55 kDa
15 kDa
K
CON
RIFSP-2
12 h
24 h
48 h
DAPI Lyso TBK1 Merge
L
TBK1 (MFI)
20 Gy
RIFSP-2
0 h 6 h 12 h 18 h 30 h 54 h
RIFSP-2
20 Gy
6 h
18 h
42 h
DAPI Lyso TBK1 Merge DAPI Lyso TBK1 Merge
M
Pearson
RIFSP-2 - 12 24 48 - - 12 24 48
20 Gy - - - - + + + + +
N
CQ
RIFSP-2
TKB1
GAPDH
1.00 0.69 1.00 0.76

Fig. S5 Influence of RIFSP-2 on STING activation and TBK1 expression in HaCaT cells

Communities classified based on the association network of the different metabolites in irradiated cells treated with or without RIFSP-2. (B) Structural character analysis of the lipid metabolites in irradiated cells treated with or without RIFSP-2. (C) Dose dependent induction of phosphorylation of STING and downstream TBK1 detected through western blotting. (D) Investigation for the potential direct interaction between RIFSP-2 and STING in vitro through surface plasmon resonance (SPR) assay. (E) Detection of the phosphorylation of STING in RIFSP-2-treated HaCaT cells through IP analysis. (F) Detection of the phosphorylation of TBK1 in RIFSP-2-treated HaCaT cells in the presence or absence of STING inhibitor, H151 through Western blotting analysis. (G) Investigation of the half-life of TBK1 in HaCaT cells treated with RIFSP-2, with administration of 5 μM CHX for indicated times. (H) Investigation on the dose (24 h) and time (15 μM) -dependent change of TBK1 expression in HaCaT cells through Western blotting analysis. (I) Detection of the TBK1 expression in RIFSP-2 (15 μM, 24 h) treated HaCaT cells, pretreated with Mg132(10 μM) or/ and CHX (5 μM) for 2 hours, through Western blotting analysis. (J) IP analysis for the ubiquitylation of TBK1 in HaCaT cells 24 h after 0 or 20 Gy irradiation. (K and L) HaCaT cells were pretreated with 15 μM RIFSP-2 for 6 hours and then the expression and colocalization with lysosomes of TBK1 were investigated in indicated times after 0 or 20 Gy irradiation through immunofluorescence microscopy assays based on specific antibody against TBK1 and LysoTracker staining. (M) Pearson correlation coefficient was calculated through Image J to explore the colocalization between TBK1 and

lysosomes in RIFSP-2 treated HaCaT cells. (N) HaCaT cells were pretreated with 10 nM chloroquine for 4 hours and administrated with 15 μM RIFSP-2 for 24 hours. Cell lysate were extracted and Western blotting analysis was adopted to investigate the influence of CQ on RIFSP-2 induced TBK1 degradation.

Figure. S6

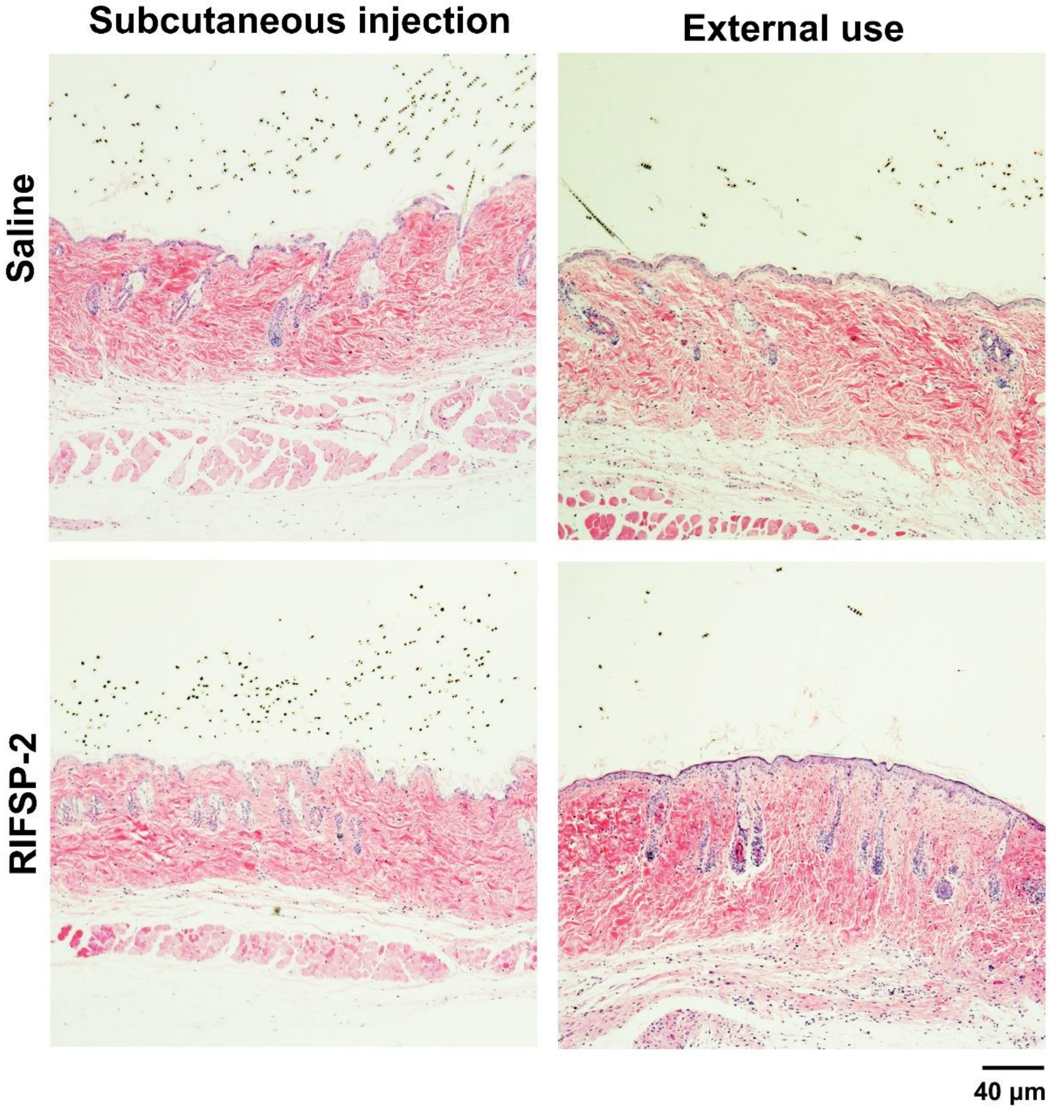

Fig. S6 Histological staining of skin from mice treated with RIFSP-2 revealed no significant pathologic differences (n = 4).

Figure. S7

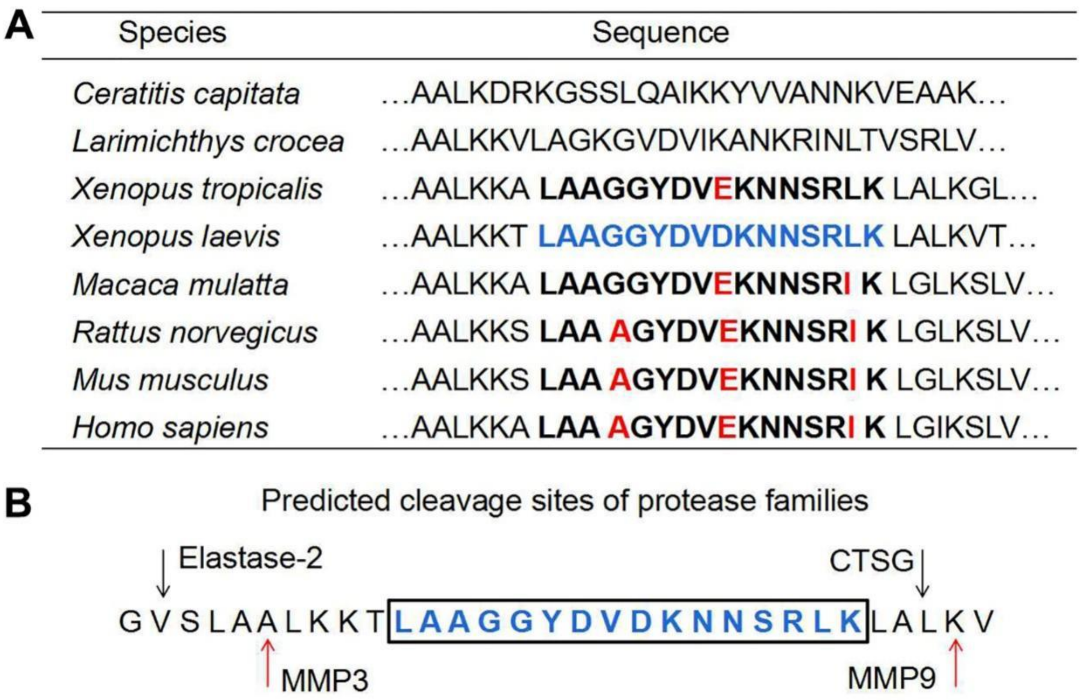

Fig. S7 (A) Homology analysis of the sequence specificity of RIFSP-2 and (B) prediction of the potential cleavage sites for this sequence using *PROSPER* (http://lightning.med.monash.edu.au/PROSPER) (37).
